# “Our desire is to make this village intestinal worm free”: Identifying determinants of high coverage of community-wide mass drug administration for soil transmitted helminths in Benin, India, and Malawi

**DOI:** 10.1371/journal.pntd.0011819

**Published:** 2024-02-06

**Authors:** Malvika Saxena, Amy Roll, Judd L. Walson, Emily Pearman, Hugo Legge, Providence Nindi, Chawanangwa Mahebere Chirambo, Angelin Titus, Jabaselvi Johnson, Elijan Abiguël Bélou, Comlanvi Innocent Togbevi, Félicien Chabi, Euripide Avokpaho, Khumbo Kalua, Sitara Swarna Rao Ajjampur, Moudachirou Ibikounlé, Kumudha Aruldas, Arianna Rubin Means

**Affiliations:** 1 The Wellcome Trust Research Laboratory, Division of Gastrointestinal Sciences, Christian Medical College, Vellore, India; 2 Department of Global Health, University of Washington, Seattle, Washington, United States of America; 3 Department of Global Health, Medicine, Pediatrics and Epidemiology, University of Washington, Seattle, Washington, United States of America; 4 The DeWorm3 Project, Seattle, Washington, United States of America; 5 Department of Disease Control, Faculty of Infectious and Tropical Diseases, London School of Hygiene & Tropical Medicine, London, United Kingdom; 6 Blantyre Institute for Community Outreach (BICO), Lions Sight First Eye Hospital, Blantyre, Malawi; 7 Institut de Recherche Clinique du Benin, Abomey-Calavi, Benin; 8 College of Medicine, University of Malawi, Blantyre, Malawi; 9 Centre de Recherche pour la lutte contre les Maladies Infectieuses Tropicales (CReMIT/TIDRC), Université d’Abomey-Calavi, Abomey-Calavi, Benin; Bernhard-Nocht-Institut fur Tropenmedizin, GERMANY

## Abstract

**Background:**

Soil-transmitted helminth infections (STH) are associated with substantial morbidity in low-and-middle-income countries, accounting for 2.7 million disability-adjusted life years annually. Current World Health Organization guidelines recommend controlling STH-associated morbidity through periodic deworming of at-risk populations, including children and women of reproductive age (15–49 years). However, there is increasing interest in community-wide mass drug administration (cMDA) which includes deworming adults who serve as infection reservoirs as a method to improve coverage and possibly to interrupt STH transmission. We investigated determinants of cMDA coverage by comparing high-coverage clusters (HCCs) and low-coverage clusters (LCCs) receiving STH cMDA in three countries.

**Methods:**

A convergent mixed-methods design was used to analyze data from HCCs and LCCs in DeWorm3 trial sites in Benin, India, and Malawi following three rounds of cMDA. Qualitative data were collected via 48 community-level focus group discussions. Quantitative data were collected via routine activities nested within the DeWorm3 trial, including annual censuses and coverage surveys. The Consolidated Framework for Implementation Research (CFIR) guided coding, theme development and a rating process to determine the influence of each CFIR construct on cMDA coverage.

**Results:**

Of 23 CFIR constructs evaluated, we identified 11 constructs that differentiated between HCCs and LCCs, indicating they are potential drivers of coverage. Determinants differentiating HCC and LCC include participant experiences with previous community-wide programs, communities’ perceptions of directly observed therapy (DOT), perceptions about the treatment uptake behaviors of neighbors, and women’s agency to make household-level treatment decisions.

**Conclusion:**

The convergent mixed-methods study identified barriers and facilitators that may be useful to NTD programs to improve cMDA implementation for STH, increase treatment coverage, and contribute to the successful control or elimination of STH.

**Trial registration:**

The parent trial was registered at clinicaltrials.gov (NCT03014167).

## Author summary

Soil-transmitted helminths (STH) are a group of three parasitic worms that infect humans through transmission of contaminated soil. An estimated 1.5 billion people world wide are infected, with the highest prevalence reported in sub-Saharan Africa, Asia, and South America. STH may cause gastro-intestinal morbidity that is known to be associated with malnutrition in children and accounts for 2.7 million disability-adjusted life years annually. The World Health Organization strategy is to control morbidity through the periodic treatment of albendazole or mebendazole of at-risk people in endemic areas, namely pre-school and school-aged children, women of reproductive age, and adults in certain occupations that pose a high-risk for transmission. Adult populations not treated may continue to act as reservoirs for STH. Modeling studies have shown that transmission of STH may be interrupted through implementation of community-wide MDA (cMDA) that targets at-risk and adult populations. Studies on cMDA for STH to assess morbidity or elimination are limited. This is a mixed-methods implementation science analysis on implementation of the DeWorm3 community cluster randomized controlled trial that aims to determine the feasibility of interrupting STH in Benin, India, and Malawi. Qualitative focus group discussions and quantitative analysis of census and coverage survey data aims to identify barriers and facilitators to implementing cMDA and compares results between high- and low-coverage clusters across the three countries. The findings may support the development of STH program guidelines and delivery strategies.

## Background

Soil-transmitted helminths (STH) affect 1.5 billion people globally and account for an estimated 2.7 million disability-adjusted life years lost annually in endemic countries [1]. The 2017 World Health Organization (WHO) STH guidelines recommend control of STH-associated morbidities via annual or biannual deworming of most at-risk populations, including pre-school and school-aged-children and women of reproductive age (15–49 years), with a goal of achieving a low (<2%) proportion of moderate and heavy intensity STH infections [2,3]. In many settings, adults serve as reservoirs leading to re-infection of at-risk populations and as a result, deworming programs may need to be continued for the foreseeable future to maintain morbidity control [4–6].

Recent studies seek to understand if interruption of STH transmission is possible through expanding deworming programs to treat all age-groups using community-wide mass drug administration (cMDA), where all community members living in an endemic area are treated with deworming medications [7,8]. The Tumikia trial in Kenya presented evidence that cMDA was associated with more rapid declines and intensity of STH prevalence and was shown to be equitable in coverage and effects [8]. To achieve declines in prevalence and elimination of STH infections, a modelling study showed cMDA programs will likely need to achieve high-treatment coverage (≈90%) [6]. As a result, it is necessary to identify the determinants of successful cMDA delivery in endemic communities [8].

Evidence from STH and other neglected tropical disease (NTD) programs indicate that MDA coverage is driven by the perceived acceptability of treatment, including demand for treatment; trust in implementers; socio-demographic factors (e.g., age, income); and programmatic factors (e.g., distribution timing; distributor training and motivation, adequate supplies, locally adapted community engagement activities) [9–15]. However, there is limited evidence on how drivers of coverage differ between areas that achieve high- and low-coverage cMDA. In fact, one study demonstrated no consistent differences in factors affecting coverage across areas [16].

A shift from school-based to cMDA programs will require significant changes to planning and implementation of deworming programs and an understanding of community perspectives to optimize delivery. The objective of this study is to identify determinants of cMDA coverage, specifically by comparing areas that achieved high and low cMDA coverage in the context of a community cluster-randomized trial in Benin, India, and Malawi.

## Methods

### Ethics statement

This study has been reviewed and approved by the Institut de Recherche Clinique au Benin through the National Ethics Committee for Health Research (002-2017/CNERS-MS) from the Ministry of Health in Benin, The London School of Hygiene and Tropical Medicine (12013), The College of Medicine Research Ethics Committee (P.04/17/2161) in Malawi, and Christian Medical College, Vellore, India (10392 [INTERVEN]). The study was approved by the Human Subjects Division at the University of Washington (STUDY00000180). The parent trial was registered at clinicaltrials.gov (NCT03014167). All adult participants provided their informed consent. Parents and caregivers (a person responsible for the care of the child who is not the parent) of child participants provided written consent and children provided written assent to participate. All participants and caregivers were assured that there was limited risk of harm from participation in this study, and that they were free to withdraw at any point.

This study used a convergent mixed-methods (QUAL+quant) design in which qualitative and quantitative data were collected in parallel, analyzed separately, and merged for integrated interpretation. We used the Consolidated Framework for Implementation Research (CFIR) to guide data collection and analysis. We collected qualitative data via community-level focus group discussions (FGDs) with local leaders, adult men and women, and children. Quantitative data were collected via routine DeWorm3 Project data collection activities, including a baseline census, routine drug distribution records, and coverage surveys.

### Conceptual framework

The CFIR informed the semi-structured FGD question guides, codebook development, and analysis [17–19]. The CFIR is a meta-theoretical determinants framework of 39 constructs that are associated with effective implementation divided into five thematic domains: inner setting, outer setting, process, individuals involved, and intervention characteristics [17]. The CFIR is a commonly used framework in implementation research to understand the effectiveness of implementation strategies and to guide the investigation of the multi-level factors that influence implementation across clusters [20]. Previous studies have used the CFIR to understand facilitators and barriers to NTD MDA uptake in Malawi and Zambia [21,22].

### Study aims and objectives

The DeWorm3 Project is a hybrid community-based cluster randomized trial that combines clinical and implementation outcomes with the objective of rapidly translating study findings into evidence that is useful to NTD programs [23,24]. The DeWorm3 trial includes forty clusters in each site (Benin, India, and Malawi), with twenty clusters randomized to receive biannual cMDA of albendazole and twenty to standard-of-care school-based deworming [23]. Implementation research takes place at project baseline, midline, and endline. Secondary implementation science aims include determining the acceptability, feasibility, and cost-effectiveness of cMDA, as well as contextual factors that influence the DeWorm3 trial outcomes, such as drivers of treatment coverage [24]. This qualitative analysis took place halfway through the intervention period (after three rounds of cMDA) in 2018–19. The analysis seeks to systematically identify and assess implementation-related barriers and facilitators to cMDA, and how these barriers and facilitators vary across clusters achieving high- and low-treatment coverage. In each site, two clusters with the highest- and lowest-average cMDA coverage, a total of twelve participating clusters, were selected to participate (Table 1).

**Table 1:** Overview of study sites.

Sites		Mean treatment coverage—High-coverage clusters		Mean treatment coverage—Low-coverage clusters	
**Benin**	Commune of Comè	**Cluster 4**	**Cluster 37**	**Cluster 17**	**Cluster 26**
		93.0%	93.7%	80.1%	80.2%
**India**	Ranipet district (formerly Vellore) and Tiruvannamalai District, Tamil Nadu	**Cluster 17**	**Cluster 34**	**Cluster 2**	**Cluster 13**
		94.2%	96.4%	91.7%	91.9%
**Malawi**	Mangochi District	**Cluster 30**	**Cluster 40**	**Cluster 3**	**Cluster 14**
		86.2%	85.8%	71.7%	78.1%

### Participant selection for qualitative data collection

In the twelve selected clusters, separate FGDs (four FGDs per cluster) were conducted with local leaders, men, women, and children ages 12–15 in Benin and Malawi and ages 10–15 in India. Inclusion criteria for men, women, and local leaders were age older than 16 years, did not already participate in another DeWorm3 FGD, and were not engaged in the DeWorm3 study, such as local drug distribution volunteers. The number of participants in each FGD ranged between 5–10 individuals from each demographic stratum who agreed to participate in person. Only one individual per household was included in the FGDs

The DeWorm3 field team and supervisors used the study census database to randomly select or purposively sample and recruit participants. Participants were randomly selected in Benin and were purposively selected in India among those who lived within 5 kilometers of the interview site given significant access challenges for in-person FGDs. In Malawi, one village from each of the four selected clusters was randomly selected, and the participants were purposively selected from the village.

Village leaders include administrative (e.g., village chiefs) and religious leaders (e.g., Sheikhs) identified by the study team and villages and were purposively selected by the study team. Participants were approached and recruited by the DeWorm3 research team (field workers and field supervisors).

### Data collection

We hypothesized that 32 of 39 CFIR constructs influence the implementation climate of cMDA and seven non-CFIR constructs were identified *a priori* to develop a semi-structured question guide to explore facilitators and barriers [see S1 and S2 Appendices]. A common guide was adapted and translated within each site for cultural appropriateness and iteratively adapted as necessary. Study teams pilot tested interview guides in Benin and Malawi with different populations and minor adaptations were made accordingly. In Benin, a question was added to collect contextual information on previous participation in vaccination programs. During FGDs, interviewers made some additional adaptations to restate or clarify the meaning of questions, including simplifying language during FGDs with children.

FGD facilitators were trained on data collection best practices. FGDs were conducted in private locations with a facilitator and a notetaker. Participants provided written consent before FGDs began. Parents/caregivers of participating children provided written consent while children ages 10–15 provided written assent, in accordance with local ethical review requirements. Following consenting and assenting procedures, 48 FGDs were audio recorded. Audio files were transcribed in the local language and translated into English. Quality checks of complete transcripts were performed in India and Malawi to compare audio to transcripts, except in Benin where four random spot-checks of 1 minute each were performed to compare audio to transcripts.

### Qualitative analysis

A mix of deductive and inductive coding was used, with an initial codebook developed based on CFIR constructs and 11 non-CFIR codes with contextualized definitions [see S2 Appendix]. The study adhered to the consolidated criteria for reporting qualitative research (COREQ) [see S3 Appendix]. During data analysis, three inductive codes were needed to describe nuances in community member behavior. Codes informed by the Theory of Planned Behavior (TPB) were developed as child codes of the CFIR construct “knowledge and beliefs”. The TPB proposes that behavior change is predicated on (1) an individual’s attitude toward the behavior (attitudes); (2) perceived social expectations to engage in the behavior (subjective norms); and (3) perceived ability to perform the behavior (perceived behavioral control) [25].

One coder was based in India, one in Benin, two in Malawi, and one at the University of Washington (UW), Seattle. Two primary coders (one site-level and one UW) independently coded each transcript in Atlas.ti (8.4.5) with a third coder designated as the “tiebreaker” when consensus was not reached between primary coders. Individually coded projects were combined into master projects for the consensus process and for case-memo development. Coders met via international conference calls for weekly consensus meetings to revise code definitions, split codes that were too large, inductively add codes, and discuss where applied codes diverged. Three case memos, one per site, were developed to make comparisons across high-coverage clusters (HCCs) and low-coverage clusters (LCCs).

**Fig 1:**
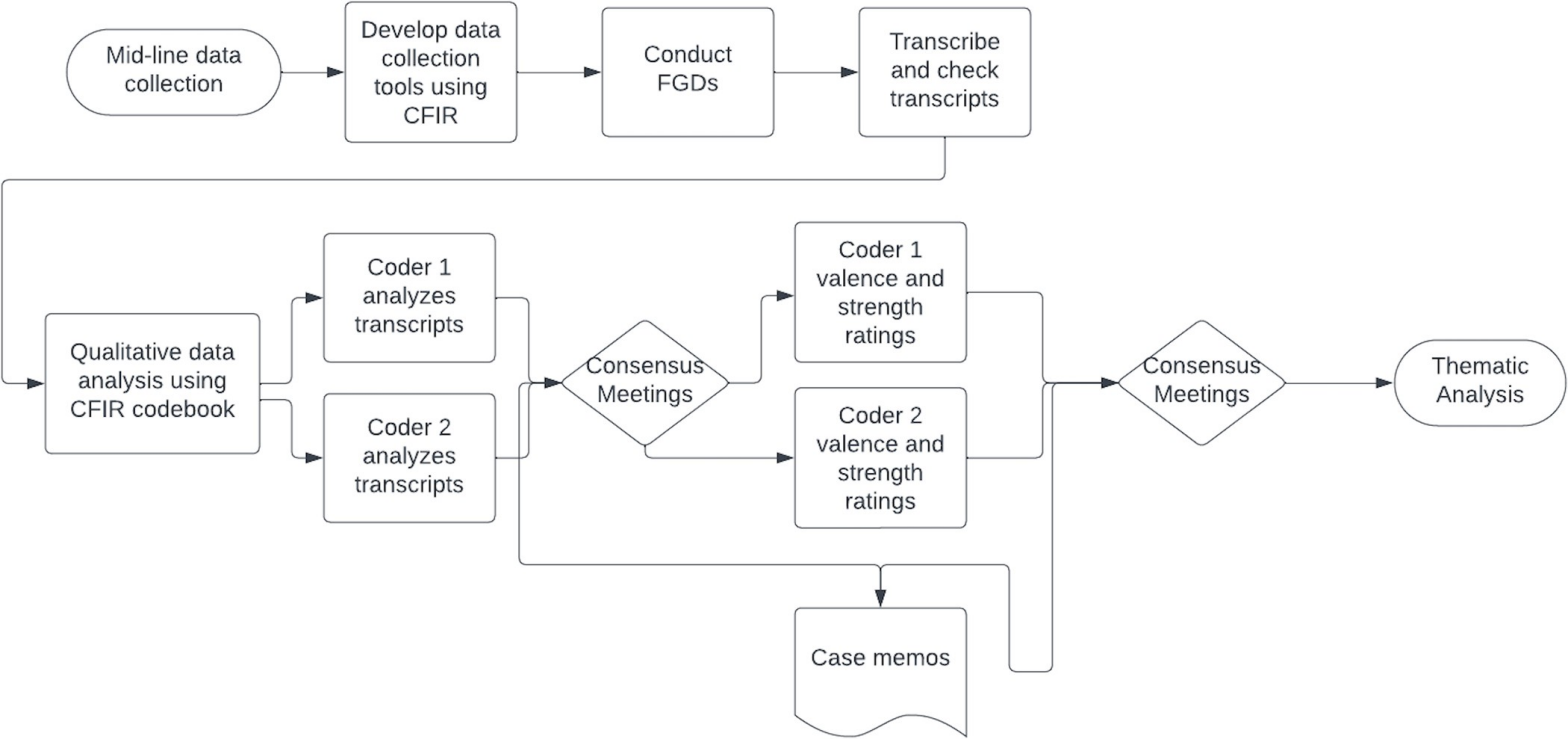
Schematic of qualitative analysis. Process used by the DeWorm3 team to collect, code, and analyze qualitative data.

The primary assumption driving this analysis was that determinants of coverage will differ between HCCs and LCCs and, as a result, different CFIR constructs will emerge as most influential in each setting. Thus, a rating process was used, developed by the original CFIR authors, and adopted across other studies [18,26,27]. Following consensus, coders separately rated each construct within an FGD to determine valence (positive, neutral, or negative influence) and strength (weak or strong influence) on the primary outcome of high coverage. Valence and strength ratings were evaluated on a scale of -2 to +2 [see S4 Appendix] [28]. For example, constructs which respondents described as strongly negatively influencing cMDA coverage with specific examples received a -2 score. To reduce bias, two coders rated valence and strength of constructs separately. Consensus meetings were held to compare ratings and discuss variation in ratings and to determine a final rating for each construct by population group in each cluster using an excel-based rating matrix. If consensus was not reached, a third coder acted as a tiebreaker. Constructs with minimal associated data were not rated. Forest plots were prepared in R (v 4.1.0) to present and highlight variability in the average and range of strength and valence scores by HCCs and LCCs by site [see S4 Appendix]. The qualitative data analysis process is depicted in Fig 1.

### Quantitative data

Quantitative data were collected during the same period (after the first three rounds of cMDA) and from the same twelve clusters. Quantitative data sources included: (1) annual censuses conducted by DeWorm3 to enumerate the population, (2) routine treatment register data completed during cMDA, and (3) post-MDA community coverage surveys at random samples of households to validate coverage and identify treatment preferences. More information about data collection activities can be found elsewhere [16]. All quantitative data were cleaned and analyzed in SAS. Frequency tables summarizing average cMDA coverage for HCCs and LCCs are presented [see S5 Appendix]. When relevant, quantitative data were presented alongside qualitative data in a joint display to identify points of convergence in findings.

## Results

The analysis includes qualitative data from 48 FGDs, in which 326 people participated in FGDs (Table 2). Fifteen people refused to participate in Benin and none in India and Malawi. The number of participants in the surveys varied by survey type, and total participation can be found in S5 Appendix.

**Table 2:** Total number of FGD participants by stakeholder group.

Stakeholder category	Benin	India	Malawi
**Men**	**35**	**21**	**21**
**Women**	**37**	**20**	**24**
**Children**	**37**	**23**	**24**
**Village Leaders (Men)**	**29**	**27**	**17**
**Village Leaders (Women)**	**6**	**0**	**5**
**Total**	**144**	**91**	**91**

**Fig 2:**
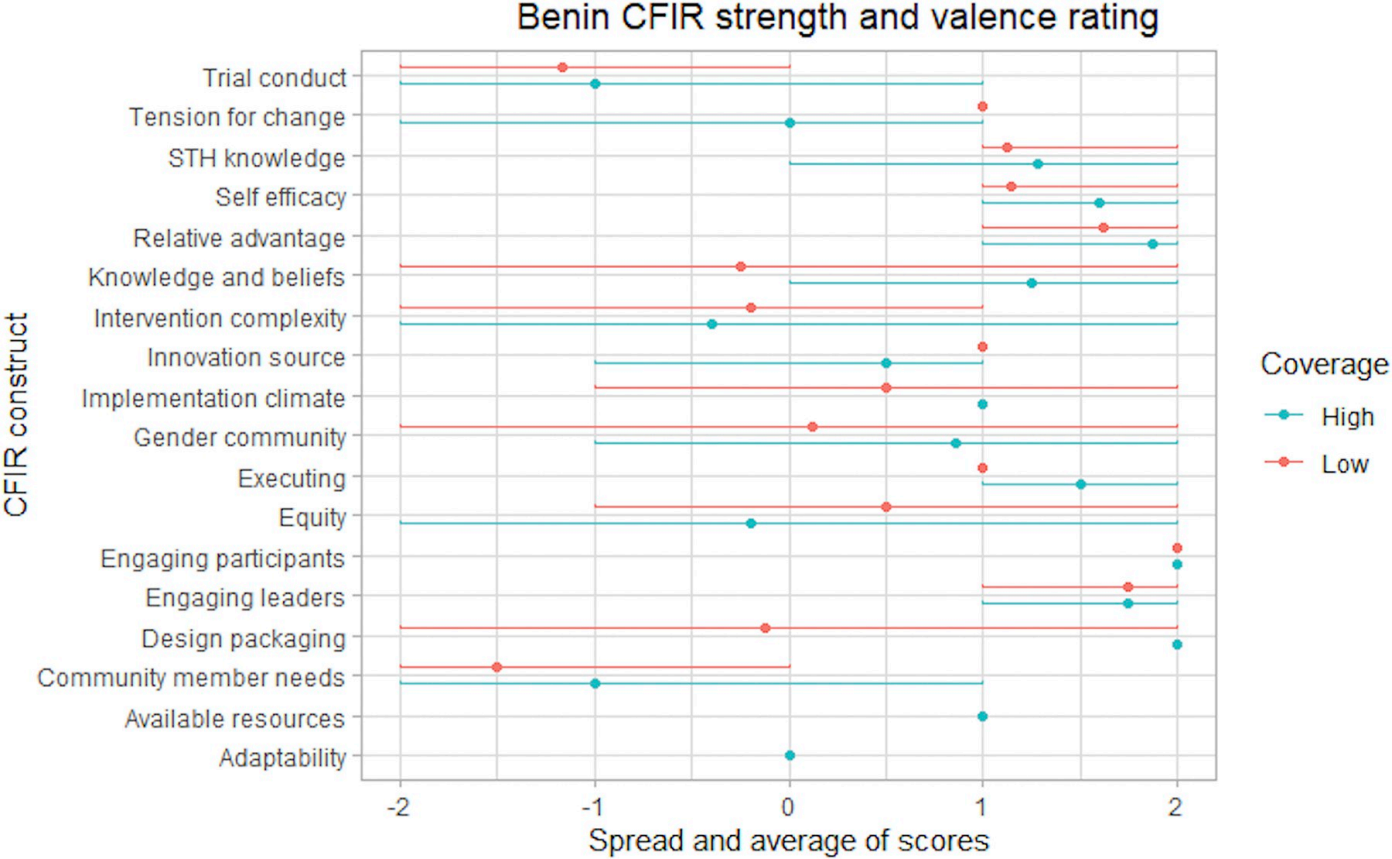
Benin CFIR strength and valence rating. Summary of strength and valence scores applied to CFIR constructs and non-CFIR codes in Benin FGDs. Excluded are constructs with limited discussion or minimal data (e.g., referenced in only one FGD by one individual). Strength and valence scores are presented as averages (dot) and ranges from the minimum to maximum (line). A dot without a line indicates consistent scores across FGDs.

**Fig 3:**
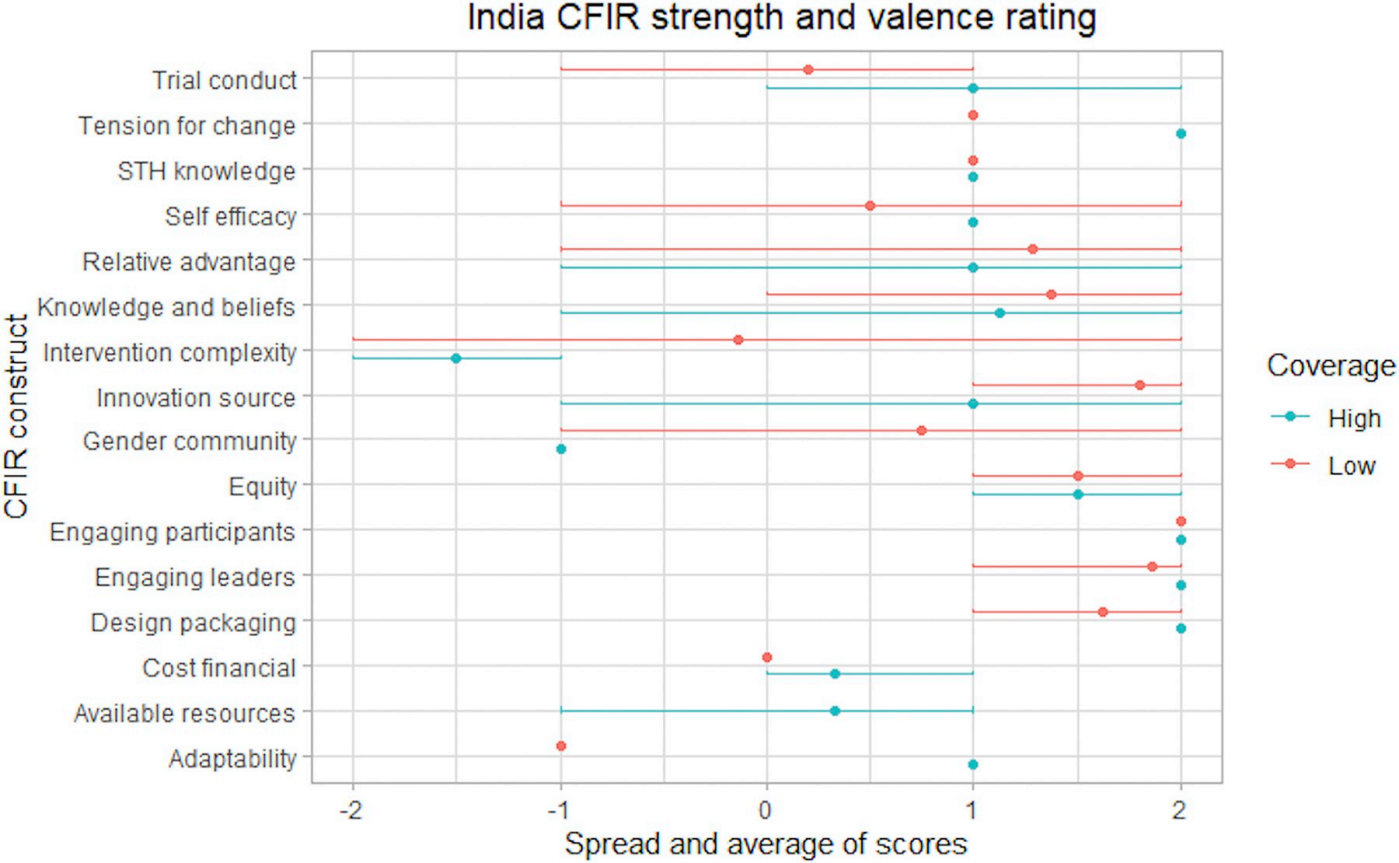
India CFIR strength and valence rating. Summary of strength and valence scores applied to CFIR constructs and non-CFIR codes in India FGDs. Excluded are constructs with limited discussion or minimal data (e.g., referenced in only one FGD by one individual). Strength and valence scores are presented as averages (dot) and ranges from the minimum to maximum (line). A dot without a line indicates consistent scores across FGDs.

**Fig 4:**
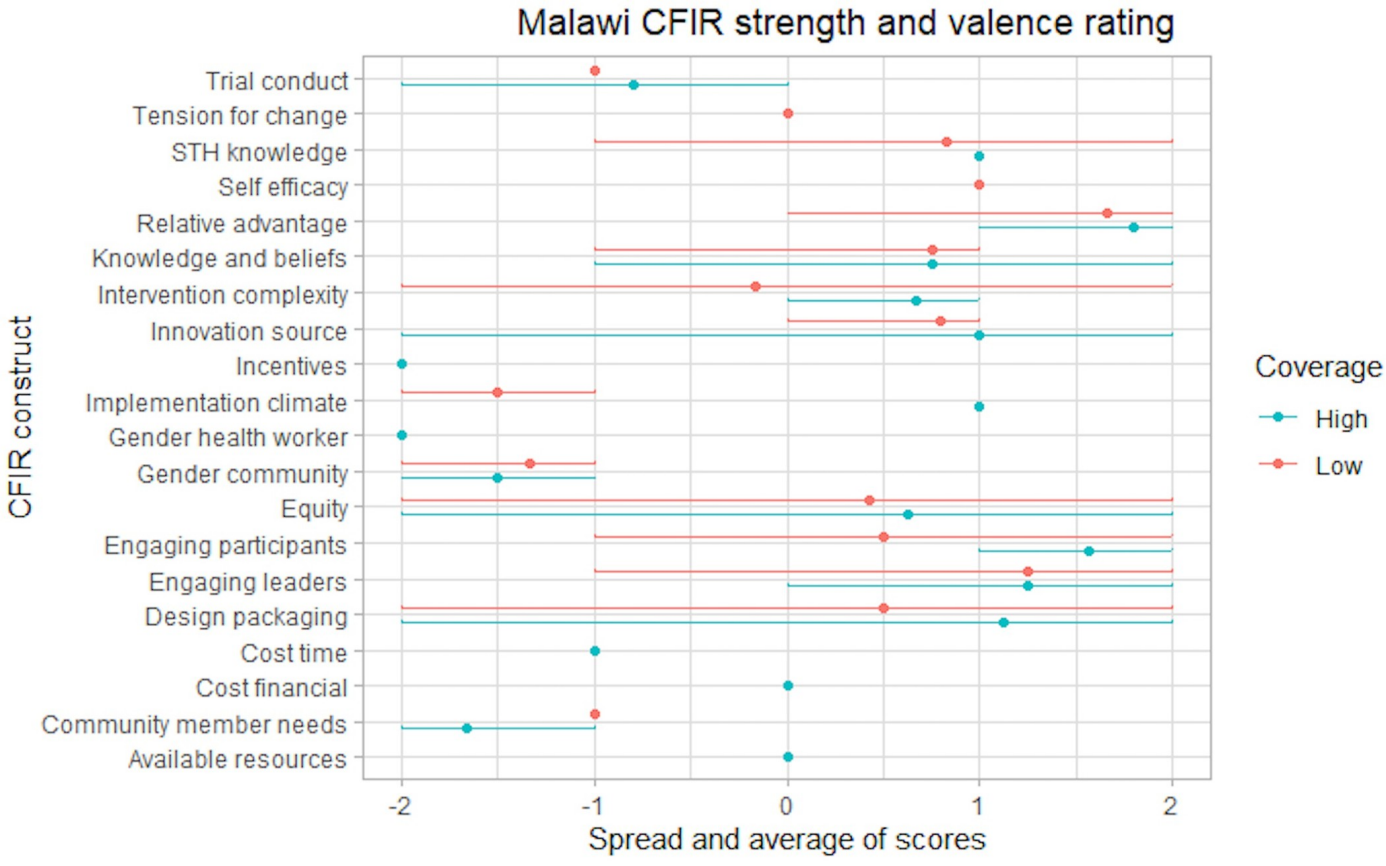
Malawi CFIR strength and valence rating. Summary of strength and valence scores applied to CFIR constructs and non-CFIR codes in Malawi FGDs. Excluded are constructs with limited discussion or minimal data (e.g., referenced in only one FGD by one individual). Strength and valence scores are presented as averages (dot) and ranges from the minimum to maximum (line). A dot without a line indicates consistent scores across FGDs.

The analysis included 23 CFIR constructs and six non-CFIR codes that were evaluated for valence and strength. The study identified 11 constructs that differentiated between HCCs and LCCs, indicating that they are potential drivers of coverage. Results fell into four overarching themes: (1) participation is influenced by the perceived trustworthiness of cMDA programs, (2) willingness to participate in cMDA is influenced by perceptions of convenience, (3) perceptions of cMDA are shaped by overarching community acceptability over time, and (4) individual participation is influenced by existing gender dynamics in communities. Key facilitators, barriers, and divergence between HCCs and LCCs are described within each theme. Corresponding quantitative data were available for nine of the 14 sub-themes. Quantitative and qualitative data converged between four and diverged between five sub-themes. The key themes are summarized in S6 Appendix, challenges and opportunities to achieve high cMDA coverage are outlined in Table 3, and summary statistics are presented in S5 Appendix. The CFIR ratings are presented in Figs 2–4 and present the average strength and valence rating (point) and the minimum and maximum rating scores (line). In some cases the construct or code was not discussed in any of the FGDs or there was minimal data (e.g., referenced by one individual) resulting in some variation between countries in constructs rated. There were also some cases where the construct or code was only discussed in the high or low coverage cluster.

### Individual participation is influenced by the perceived trustworthiness of cMDA programs

Trust in cMDA was a key determinant in achieving high coverage cMDA. Facilitators included perceived effectiveness of cMDA in treating STH infections (*relative advantage*), perceived safety of deworming (*design quality and packaging*, *innovation source*), and engagement strategies that collaborated with local leaders to build credibility of cMDA (*engaging leaders and participants*, *design quality and packaging*). Key barriers included safety concerns (*patient needs and resources*) and negative perceptions of some trial activities (non-CFIR construct *trial conduct*). Perceptions of trust diverged between HCCs and LCCs, particularly in Benin, driven by mistrust of directly observed therapy (DOT) (*design quality and packaging*), beliefs in myths and rumors about cMDA, and perceptions of previous public health programs (*knowledge & beliefs–attitudes*).

#### Facilitators

Adults in all sites perceived themselves to be at risk for STH infection and believed it was necessary to receive treatment. Participants reported a strong preference for community-wide door-to-door deworming that treated individuals of all ages, rather than school-based deworming targeting children only.

“The distribution of the intestinal medicine both to school-going children and adults is a very welcome idea. It shows that the disease is contagious and when groups of people are not receiving while others are receiving it will not be eradicated, but now we are sure that the disease will be reduced…it will help to eradicate the disease once for all.” Respondent 5, Local Leaders (HCC), Malawi

In Malawi, familiarity with the treatment drug (albendazole) from previous lymphatic filariasis (LF) cMDA programs made it easier for participants to trust the STH cMDA program.

“We trusted [DeWorm3] because it was the same medicine similar to what we received in the past and we will be trusting them when they come again.” Respondent 6, Women (LCC), Malawi

cMDA was perceived to be safer and thus more trustworthy if the program was associated with a non-governmental organization (NGO) rather than a government program in India and Malawi.

“If it is government tablet, they will eat with fear…most of them will not eat it. If it is private everyone will eat with trust, since it is government tablet there is no chance for them to eat.” Respondent 4, Men (HCC), India

cMDA was more trustworthy when the program collaborated with local leaders to educate people and encourage participation in cMDA. When viewed as authentic collaborators, leaders were able to effectively communicate and ensure alignment between cMDA program goals and communities.

“[A]nother reason why most of the people are interested in taking part in this program is because the link we have between the leaders and an organization when implementing the program in place, which is different in the past where an organization was just implementing the program without proper collaboration with the leaders. But now the leaders and an organization are working together for the same goal. People understand and are interested to take part in these programs.” Respondent 5, Local Leaders (HCC), Malawi

While local leaders were engaged in mobilization in all sites, quantitative coverage survey data indicate that an average of only 5% (Benin), 0.2% (India), and 14% (Malawi) of survey participants were made aware of cMDA by local leaders. Qualitative FGDs with local leaders show that they may have a more important role in collaborating with health workers to encourage non-participants specifically to reconsider taking treatment in some cases.

“When she refused, a traditional religious leader came and told her that no one should refuse treatment. Whatever your religion, if you are sick, you must do the treatment by taking drugs or injections.” Respondent 1, Men (LCC), Benin

More often survey participants reported that they heard about the program from family and friends: 68% (Benin), 49% (India), and 33% (Malawi); and/or from CDDs/teachers: 39% (Benin), 10% (India), and 43% (Malawi). Survey participants often stated that they had more confidence in cMDA when they learned about it from someone they knew. The qualitative data did however lack examples of respondents describing learning about cMDA from friends and family.

“They [CDDs] put up drama before giving, after that some people are correcting themselves. So, now they say that the toilet should be used for defecation; should wear footwear and go. Many people are correcting because of this.” Respondent 1, Women (HCC), India

Qualitative data indicate that people would have more confidence in cMDA if they were informed by health center staff, like doctors. However, on average, survey participants rarely learned about cMDA from this group: 0.9% (Benin), 3% (India), and 17% (Malawi).

“In my opinion, many of us like to go to the hospital…In other words, we trust doctors more than the mere Community Drug Distributors” Respondent 2, Children (HCC), Benin

#### Barriers

Across HCCs and LCCs in Benin and Malawi, participants reported that health workers advised participants to take deworming tablets after meals. Participants believed that cMDA needed to be delivered alongside food.

“It is not possible for a person to take medicine without eating. Even the drugs we receive from the hospital, they advise us to take after eating food…the medicine should be distributed in our homes and to advise us to take it after children and even parents have eaten food.” Respondent 4, Men (HCC), Malawi

DeWorm3 trial activities include the annual collection of stool samples for STH prevalence surveys. Community members in all three sites reported stigma and mistrust around stool collection that negatively affected perceptions of cMDA.

“People may call the officials who will be coming to distribute the medicine bad names like feces officers…What should be done is to visit people and inform the importance of the program. But to the contrary people were just told to give their stools without being told the importance of it.” Respondent 1, Local Leaders (HCC), Malawi

#### Findings that differentiate between high- and low-coverage clusters

CDDs were instructed to observe participants taking the deworming tablet when possible to ensure drug compliance. Some participants reported that without DOT, people would not have taken albendazole.

“They make us eat in front of their eyes. If it is not so, we do not eat it, we don’t care about it…So, it is an advantage.” Respondent 1, Men (LCC), India

However, in Benin, DOT was perceived as less favorable in LCCs (Fig 2: design quality and packaging). Participants in LCCs described feeling forced to take the treatment during cMDA, especially if they felt unprepared to participate (e.g., if they had not eaten beforehand). These findings converged with MDA treatment data, which indicate that more survey participants (86%) in HCCs were treated via DOT compared to survey participants (69%) in LCCs in Benin. Further, there was a higher rate of tablets being left with survey participants who were present but refused DOT in LCCs versus HCCs (4% vs 0.1%).

“The medication should not be required to be taken on the spot in front of the drug distributors. If the person asks you to leave the medication for them, we should accept.” Respondent 3, Local Leaders (LCCs), Benin

Participants across all sites reported the presence of myths and rumors about cMDA. In Benin, negative rumors about deworming treatment had a greater impact on acceptance of cMDA in LCCs (Fig 2). Negative attitudes were fueled by rumors that cMDA was actually distributing birth control tablets, or potentially dangerous drugs.

“But we heard some rumors. Some say they have refused treatment because they don’t know if it is to kill people.” Respondent 4, Women (LCCs), Benin

However, coverage surveys indicate that an average of 99.7% (HCCs) and 99.6% (LCCs) of survey participants in Benin self-reported that they accepted treatment of albendazole.

Participants in India and Malawi, and participants in HCCs in Benin reported more positive experiences with previous community health programs, like vaccination campaigns. These prior experiences positively influenced their perceptions of cMDA safety and willingness to participate.

“Like measles vaccination, none of this [deworming program] is bad. These initiatives are aimed at saving ourselves and we thank the promoters.” Respondent 4, Men (HCCs), Benin

In contrast, participants in LCCs in Benin reported more community resistance to previous school-based MDA and vaccination programs. This was due to reports of negative side-effects following treatment that led people to resist the new STH cMDA program.

“[T]here was a drug distribution for the kids at school and some kids got sick after taking the medication. It is because of these illnesses that parents refuse and prevent their children from taking the medicine.” Respondent 2, Children (LCCs), Benin

### Individual willingness to participate in cMDA is influenced by perceptions of convenience

Successful delivery of cMDA was dependent upon community perceptions that it is a convenient delivery method. Some respondents reported that cMDA removed barriers to accessing treatment (*design quality and packaging*, *non-CFIR code behavioral control*). However, a key barrier was participant dissatisfaction with implementation plans that favored in-person treatment for absent family members (*intervention complexity*). Perceptions of convenience diverged between HCCs and LCCs in Malawi, driven by challenges related to delivery timing and frequency (*intervention complexity*).

#### Facilitators

Across all sites, participants reported that door-to-door delivery reduced time and costs associated with adults proactively seeking deworming treatment from health centers or private pharmacies. In Malawi, participants reported cMDA increased accessibility, particularly for the elderly and families that cannot afford to send their children to school. In Benin and India, several participants reported they previously bought albendazole for their children but believed that cMDA made it possible to now deworm all family members.

“The door-to-door initiative has helped those who can’t afford to reach to schools and hospitals or even where such drug distribution events are happening especially the elderly and children…” Respondent 5, Men (HCCs), Malawi

Findings are validated by coverage surveys, which indicate that an average of 97% (Benin), 86% (India), and 77% (Malawi) of survey participants preferred door-to-door distribution over school-based or centralized distribution. Qualitative data helps contextualize people’s preference for door-to-door distribution in that it reduces time, cost, and access barriers.

#### Barriers

Participants in Benin and Malawi were dissatisfied that CDDs could not leave drugs with family members of individuals absent at the time of distribution. They perceived the implementation plan to be unnecessarily complicated and suggested medicine should be left with family members instead of CDDs returning to treat absent individuals.

“There is something that the agents do that is not right, and it is that I, the father, have taken some, my wife too, my children were not present at home; when I ask to be given their share so that I could give it to them when they return, they refused, saying that the children should be drinking it in front of them…The agents must not leave with the drugs.” Respondent 2, Men (HCCs), Benin

MDA treatment data indicate that most survey participants who received treatment were treated via DOT and only a small proportion of survey participants could not be reached by DOT after three visits: 0.2% (Benin), 3% (India), and 0.6% (Malawi).

#### Findings that differentiate between high- and low-coverage clusters

Some participants in LCCs in Malawi indicated that cMDA was disruptive to their daily activities as it was conducted when people were unavailable due to economic activity (Fig 4). This was a deviant finding.

"[A] lot of people leave their homes early in the morning to do business, work in the fields such that when the people come, they don’t find them.” Respondent 2, Women (LCCs), Malawi

Even when participants were not present for treatment, participants in Benin, India, and HCCs in Malawi were reassured that repeat visits would provide another opportunity to participate.

“The present program was different because when a person was not present at his/her house, they were coming back and give them the drugs to take in instantly.” Respondent 6, Local Leaders (HCCs), Malawi

Coverage surveys indicate that an average of 16% of survey participants in HCCs thought that MDA was delivered at an inconvenient time compared to 5% of survey participants in LCCs in Malawi. Further, most survey participants in Malawi were reached during their first visit: 89% (HCCs) and 83% (LCCs).

### Individual perceptions of cMDA are shaped by overarching community acceptability over time

#### Findings that differentiate between high- and low-coverage clusters

Participation in cMDA was influenced by an individual’s perception of cMDA acceptability within their community. In Malawi, community-level receptiveness to cMDA diverged in HCCs and LCCs (*implementation climate*) (Fig 4). However, some participants reported that as familiarity with community campaigns grew over time, so did demand for cMDA in HCCs in Malawi and across clusters in India and Benin.

In India, participants indicated that community trust is built over several years, and only after communities have a positive experience participating in cMDA.

“After eating, if it is good, they will eat the next time. It will take one-two-three years for us to develop a trust when you are introduced to us.” Respondent 3, Local Leaders (LCCs), India

Participants in Benin and in HCCs in Malawi reported that individuals were more likely to participate when cMDA was perceived to be highly socially acceptable, specifically when individuals observed their neighbors participating in cMDA.

“Most of the people have received the intestinal medicine this time because they observed their friends who received last time, they are getting better than before, as a result, they were admiring their friends to follow suit in receiving the medicine.” Respondent 6, Local Leaders (HCCs), Malawi

Conversely, some participants in LCCs in Malawi deviated and intentionally avoided distributors after the first round and there were reports of people no longer wanting to take the drugs.

“During the first MDA, a lot of people drunk the medicine…But this second distribution, a lot of people were escaping because they knew the people who were distributing the medicine so they could run away from them.” Respondent 2, Women (LCCs), Malawi

Treatment data indicate that the average proportion of survey participants treated by DOT increased between MDA round 1 and round 3 in both HCCs and LCCs in Malawi; 67% were treated by DOT in round 1 versus 83% in round 3 in HCCs and 64% in round 1 versus 77% in round 3 in LCCs. Qualitative data provide context that trust in a program may take years to develop.

### Individual participation is influenced by participants’ sex

Respondents reported that cMDA engagement and delivery strategies reached women more successfully than men (non-CFIR code *gender-community*). However, in Benin, household decision-making by women differentially influenced participation in HCCs and LCCs (Fig 2).

#### Facilitators

In India and Malawi, participants reported that cMDA successfully reached women. Women were more likely to be home during distribution and to receive outreach messages. Men were more likely to be away from home or migrating for work. Qualitative findings are corroborated by MDA treatment data that among eligible adult male survey participants, 15% (Benin), 8% (India), and 32% (Malawi), were untreated while among eligible adult women survey participants, 13% (Benin), 7% (India), and 16% (Malawi) were untreated, with the greatest difference in Malawi.

“Some men may be missed out because they will not be there at that time in the house. When it is like that, they come and give the next day. If they are not available also at that time, some people may be left out. For ladies, no one is missed out” Respondent 1, Women (HCCs), India

In Malawi, some men in LCCs reported that they did not receive communication about cMDA before or during cMDA.

“The group that received most are women. Women accepted and quickly realized the importance of intestinal medicine more than men…Most men were afraid of the unknown.” Respondent 3, Local Leaders (HCCs), Malawi

#### Findings that differentiated between high- and low-coverage clusters

Women in HCCs reported more latitude to exert decision-making authority on behalf of their household than women in LCCs in Benin (Fig 2: gender community). Women in HCCs stated that they were responsible for health-related decisions in their households. In LCCs, both women and men survey participants reported that decisions were usually made by the male heads-of-households. In Benin, there were slightly more female-headed households in HCCs (41%) compared to LCCs (39%). Qualitative findings converge with quantitative data indicating that entire households were treated during the first visit more frequently in female-headed households in HCCs (87%) compared to female-headed households in LCCs (83%) in Benin.

“If we are distributing medicine to the community and my husband says not to give it to children while I, as a woman, know that it is a medicine that has virtue, I will have my children drink it without my husband’s knowledge; I will insist on giving the medicine to children.” Respondent 9, Women (HCCs), Benin

Table 3 summarizes the implementation challenges and opportunities identified by FGD and survey participants. The study draws on suggestions proposed by participants, mainly from FGDs.

**Table 3:** Implementation challenges and opportunities for high cMDA coverage.

Challenges	Participant Suggestions
Rumors and myths about STH treatment and side effects leads to mistrust of cMDA programs	• Collaborate with local leaders to align the goals of cMDA with communities and mobilize community participation • Recruit CDDs and HSAs from the local communities • Collaborate with NGOs to build trust in government programs
Mistrust of government-run programs negatively impact people’s perceptions and acceptance of current cMDA programs	• Timely and repeated messaging on STH, drugs used, and side effects. • Endorsing media stories that encourage the social norm of compliance and safety of the drug under use
Refusal of DOT in some setting	• Improve messaging around the purpose of DOT in sensitization activities
Negative experiences with previous community health programs cause resistance towards participation in current cMDA	• Tailor engagement strategies to directly address concerns from previous experiences with past MDA and community health programs • Engage with village leaders to establish rapport between the program and communities

## Discussion

This study explored determinants (facilitators and barriers) of cMDA coverage from the perspective of community members in Benin, India, and Malawi. We identified determinants related to cMDA delivery, including reaching households at convenient times. We also identified determinants related to community demand for cMDA, including community social norms and sex dynamics. In several circumstances, these determinants presented differently in high- and low-coverage clusters, indicating that they are potential drivers of coverage. Determinants differentiating between HCCs and LCCs include experiences with previous community-wide programs, perceptions of DOT, perceptions about treatment uptake of neighbors, negative rumors and myths about cMDA treatment, and women’s agency to make household-level treatment decisions. Addressing these determinants may be particularly helpful in increasing coverage in some settings.

The demand for programs influenced coverage, particularly the perceived trustworthiness of cMDA that differentiated between HCCs and LCCs in Benin. Trustworthiness was influenced by both rumors and myths about treatment side effects and previous experience with health campaigns. In Benin, participants in LCCs reported more negative experiences with prior health campaigns, while HCCs reported positive experiences that shaped their beliefs about safety and effectiveness of health campaigns. Studies from Kenya [10,29,30] and Uganda [31] reported similar circulating rumors, including that MDA is distributing dangerous and expired medicine. Further, one study in Kenya found similar negative experiences with prior MDA influenced demand for current services [10]. Trial survey findings indicate that rumors may not have had as much of an impact on acceptance of cMDA as was indicated in the qualitative acceptability data. This suggests that the program may have been able to overcome myths and rumors to achieve high acceptance of treatment and one suggestion is to tailor outreach activities to directly address specific concerns related to negative rumors and myths identified by communities.

Participants also reported higher demand for cMDA if delivered by NGOs as opposed to governments, driven by a mistrust of government programs. Similar findings have also been reported in lymphatic filariasis programs, where community mistrust of the government persisted across generations and negatively impacted perceptions of community initiatives [32]. Previous cMDA studies of STH and LF in Kenya and Zambia reported leveraging traditional leadership structures, such as village chiefs and religious leaders, in engagement activities to increase demand for MDA [10,33]. While in this study quantitative data indicate that leaders play a minimal role in community outreach before launching cMDA, we highlight that their primary role may be adding legitimacy to campaigns. This in turn may help to address concerns about government delivery of medicines.

Participation is also strongly linked to the perceived convenience of delivery. Participants noted a strong preference for door-to-door delivery, which converged with quantitative findings. A systematic review of schistosomiasis MDA reported that home-based delivery led to higher treatment of children than school-based approaches and was perceived as more convenient [9]. Preference for door-to-door treatment in this study was also driven by favorable attitudes towards reaching vulnerable populations, including the elderly and out-of-school children, both groups who previously had limited or no access to STH treatment.

While many participants found cMDA delivery to be convenient, others found treatment overly complex, especially program guidance not to leave medicine for absent family members until three attempts to treat in-person were made. Coverage survey data suggest that survey participants who thought cMDA distribution was inconvenient and who also missed distribution may only represent a small minority of participants. Qualitative evidence speaks to the challenge of creating an adaptable and convenient delivery strategy designed to meet everyone’s needs, as described in other studies [10,34,35]. Multiple visits by CDDs for ensuring DOT may create more complexity in implementation but may be needed to avoid systematic non-participation in subgroups.

Many participants in this study found DOT to be highly acceptable. However, participants in LCCs in Benin were dissatisfied with DOT when they felt pushed to participate and when CDDs would not leave the medicine on request for absent family members. Evidence from LF programs in multiple settings indicate that DOT was both acceptable and drove high-treatment coverage [12,36–39].

While DOT is widely accepted and is often credited for driving high treatment coverage in some settings, programs will need to gain community trust in order to deliver cMDA with high coverage. In some settings, this may mean encouraging collective community action to engage in DOT. In others, where DOT has low acceptability or might compromise community trust in public health infrastructure, programs may need to identify alternative implementation plans. In both circumstances, it will be important for researchers and implementers to understand community preferences.

Lastly, participants in this study discussed supply and demand challenges related to gender and sex in cMDA participation. Other studies have similarly noted that MDA coverage is generally higher for women and that men perceive themselves to be at lower risk for STH [40,41]. Programs could consider engagement strategies that specifically target men. For example, previous LF-MDA programs reported success in reaching men through text messages and internet and radio announcements [33,42]. This study also found that women in HCCs in Benin were able to make treatment decisions on behalf of their households. Our findings suggest that more equitable healthcare decision-making between men and women within households has a positive influence on cMDA coverage. Further research is needed to determine if participation in cMDA itself has positive gender transformative consequences.

We identified demand challenges that NTD control programs that are considering cMDA can use to guide implementation. Specifically, we identified challenges to participation that may be addressed through social mobilization and communication strategies that address concerns, including around DOT, and mobilize community influencers to promote participation in cMDA.

### Limitations

There are several limitations to this analysis. First, qualitative and quantitative data were collected and analyzed separately, leaving some missed opportunities for quantitative exploration of some qualitative findings. Both the coverage surveys and the MDA registry had many individuals that were surveyed at each time point and the quantitative data did not adjust for repeated measure bias. The FGDs were recorded and conducted by organizations implementing cMDA, therefore, there is a possibility of observation bias in the qualitative data. We addressed this limitation by informing participants how anonymity of the data would be maintained and by ensuring that no field officers involved in MDA were chosen as FGD facilitators within a cluster. Additionally, the representativeness of the four clusters sampled in each site may be limited, especially in India, where the gap between HCC and LCC MDA coverage was relatively small. This may affect generalizability of findings. Coders may have introduced observation bias during assignment of valence and strength rating, which may affect interpretation of results. To address this potential bias, multiple coders, including at least one coder in each of the three countries, engaged in analysis and rating assignments, and held regular meetings to discuss and compare results to reach consensus. Lastly, this study limited gender participation to the binary of men and women and the study design did not specifically address sex and gender sensitivities. We presented results that differentiated between the sexes but were not able to provide a more gender-specific analysis. The study also has a number of strengths, including the relatively large number of participants across three very heterogenous settings, which allows for an in-depth analysis of factors affecting coverage that may affect future STH elimination programs.

### Conclusion

This convergent mixed-methods study identified and compared barriers and facilitators to achieving high coverage of a cMDA program for STH in three countries following three rounds of treatment. We identified fourteen subthemes, including six that differentiated between HCCs and LCCs. Qualitative and quantitative findings converged within four sub-themes. These findings are relevant to MDA programs attempting to improve or maintain high coverage of cMDA. For example, to increase demand for treatment programs may need to address convenience concerns around DOT to ensure maximum participation. These findings may be useful to NTD control programs and Ministries of Health to improve cMDA implementation to increase treatment coverage, and ultimately, contribute to the successful control and/or elimination of STH.

## Supporting information

S1 Appendix Qualitative interviw guide. (DOCX)

S2 Appendix Qualitative codebook. (DOCX)

S3 Appendix COREQ Table. (DOCX)

S4 Appendix CFIR construct rating rules. (DOCX)

S5 Appendix Quantiative tables (full tables). (DOCX)

S6 Appendix Summary of key themes. (DOCX)
