# Supplementary material for: “Our desire is to make this village intestinal worm free”: Identifying determinants of high coverage of community-wide mass drug administration for soil transmitted helminths in Benin, India, and Malawi
Source: PLoS Negl Trop Dis. 2024 Feb 6;18(2):e0011819. doi: 10.1371/journal.pntd.0011819 (PMC10846705; doi:10.1371/journal.pntd.0011819)
Supplement: S1 Appendix — (DOCX) [file pntd.0011819.s001.docx]

**S1 Qualitative interview guide**

| # | Question |
| --- | --- |
| I am going to ask you some questions about how community-wide MDA for worms has been delivered in your community. We hope to learn from your experiences about what has gone well, what has not gone well, and how MDA can be improved in the future. Your experiences will help us learn about how to make these programs better not only in this community, but in other communities suffering from worms as well. | |
| 1 | - What advantages are there to treating everyone in the community for STH, as compared to just treating children in schools? What disadvantages? |
| 2 | - Did you think MDA for STH was delivered appropriately in your community? Why or why not? - What would you change about the way that community-wide MDA for STH was delivered in your community? - What would you not change about how community-wide MDA for STH was delivered in your community? |
| 3 | - What barriers do community members face in participating in community-wide MDA for STH? - *Follow-up questions if more information about solutions is required: Why do these barriers exist? What could be done to overcome these barriers?* |
| 4 | - Are all community members reached with DeWorm3 MDA interventions equally well? Who is reached well and who is not reached well, and why? |
| 5 | - Do you remember the lymphatic filariasis (LF) MDA programs that used to occur in this area? What do you remember about them? *Note: If community members do not remember LF days, ask them about other community-based public health programs, such as trachoma MDA.* - Did you participate in those treatment days by swallowing the medicines given to you? Why or why not? - How has your previous experience with the LF program affected your interest in participating in the current mass treatment days? |
| 5 | - How should community members be informed about community-wide MDA or other DeWorm3 interventions before they occur? - Who do you trust to give you information about public health activities, such as community-wide MDA? |
| 7 | - What are some strategies to make sure that everyone is reached by MDA, or other community-based healthcare programs? |
| 8 | - *ONLY if there is time:* What community-based healthcare activities, other than MDA for STH, are effectively reaching people in your community? What do you like or dislike about these healthcare activities? |
