# Supplementary material for: “Our desire is to make this village intestinal worm free”: Identifying determinants of high coverage of community-wide mass drug administration for soil transmitted helminths in Benin, India, and Malawi
Source: PLoS Negl Trop Dis. 2024 Feb 6;18(2):e0011819. doi: 10.1371/journal.pntd.0011819 (PMC10846705; doi:10.1371/journal.pntd.0011819)
Supplement: S2 Appendix — (DOCX) [file pntd.0011819.s002.docx]

**S2 Qualitative codebook**

| **Construct** | **Construct definition** | **Inclusion/ exclusion for code application** | **Code** |
| --- | --- | --- | --- |
| **DOMAIN: Intervention characteristics**  *Aspects of an intervention that may impact implementation success* | | | |
| **Evidence strength & quality** | Stakeholders’ perceptions of the quality and validity of evidence supporting the belief that cMDA will have desired outcomes. | **Inclusion Criteria:**  Include statements regarding awareness of evidence, the strength and quality of evidence, the absence of evidence, or a desire for different types of evidence regarding STH transmission interruption.  **Exclusion Criteria:**  Exclude or double code (depending upon statement content) statements regarding the generation of evidence as an engagement strategy to “Engaging”. | Evidence |
| **Relative advantage** | Stakeholders’ perception of the advantage of cMDA versus an alternative solution, such as school-based deworming. | **Inclusion Criteria:**  Include statements that demonstrate that the community-wide intervention is better or worse than existing or comparable strategies/programs.  **Exclusion Criteria:**  Exclude statements that demonstrate a strong overall need for community-wide MDA or that state that school-age-MDA is unsustainable. These should be coded as “Tension for Change”. | Relative_Advantage |
| **Adaptability** | The degree to which an innovation can be adapted, tailored, refined, or reinvented to meet local needs. | **Inclusion Criteria:**  Include statements regarding the ability or inability to adapt and/or customize delivery of community-wide MDA to the context.    **Exclusion Criteria:**  Exclude or double code statements about whether or not changes to cMDA can be implemented by the MOH to “Compatibility.” Code recommendations or suggestions for improvement to “Recommendation”. | Adaptability |
| **Complexity** | Perceived difficulty of the cMDA, reflected by duration, disruptiveness, and number of steps required to implement. | **Inclusion Criteria:**  Code statements regarding the complexity of designing or delivering community-wide MDA.  **Exclusion Criteria:**  Exclude statements regarding the complexity of delivering health programs generally (beyond cMDA) within the health system climate and code to the appropriate CFIR code, e.g., difficulties related to engaging participants in a new program are coded to “Engaging.” | Intervention_complexity |
| **Design quality & packaging** | Perceived excellence in how the cMDA program is bundled, presented, and assembled. | **Inclusion Criteria**  Include statements regarding the packaging, integration, or presentation of community-wide MDA within the public health system. Recommendations and suggestions for improvement of packaging, integration, or presentation to current cMDA can be coded here.  **Exclusion Criteria** Exclude statements regarding whether or not the resources are actually present or absent and code to “Available Resources.” Exclude statements regarding recommendations that are out of scope of the current cMDA program (e.g., incorporating other public health outreach into the program) and code under “Adaptability.” | Design_Packaging |
| **Design quality & packaging (LF)** | Perceived excellence in how the cMDA is bundled, presented, and assembled. | **Inclusion Criteria**  Include statements regarding the packaging, integration, or presentation of community-wide STH MDA within LF platforms in the health system.  **Exclusion Criteria** Exclude statements regarding whether or not the resources are actually present or absent and code to “Available Resources.” | Design_Packaging_LF |
| **Cost (financial)** | Costs associated with implementing cMDA. | **Inclusion Criteria:**  Include statements related to the monetary cost of community-wide MDA and its implementation.  **Exclusion Criteria:**  Exclude or double code statements about whether or not financial resources are available to “Available Resources.” | Cost_financial |
| **Cost (opportunity)** | Opportunity costs of cMDA including increases in time. | **Inclusion Criteria:**  Include statements related to the opportunity costs of community-wide MDA (ex. time) and its implementation.  **Exclusion Criteria:**  Exclude or double code statements about whether or not resources are available to “Available Resources.” | Cost_time |
| **Innovation source** | Perception of key stakeholders about whether cMDA is externally or internally developed. | **Inclusion criteria:** Include statements where stakeholders are discussing the development and delivery of cMDA, and who is involved with the intervention development, as well as whether there are different perspectives on the intervention because it is a research intervention versus an MOH program.  **Exclusion Criteria:**  Exclude or double code statements related to participation in the intervention to “engaging participants”. | Innovation_source |
| **DOMAIN: OUTER SETTING**  *Features of external context or environment that may impact implementation* | | | |
| **Patient needs and resources** | The extent to which the needs of those served by cMDA (e.g., targeted community members), as well as barriers and facilitators to meet those needs, are accurately known and prioritized by the organization. | **Inclusion Criteria:**  Include statements demonstrating presence or absence of the interviewee’s awareness of the needs of community members. Coders may be able to infer about awareness based on statements about: 1. Perceived need for community-wide MDA to improve community member health; 2. Barriers and facilitators of community members to participating in MDA; 3. Participant feedback on the innovation, i.e., satisfaction with the program. In addition, include statements that capture whether or not awareness of community member needs influenced the implementation or adaptation of the intervention. Include statements when community members describe their health needs beyond cMDA and whether or not community members believe the implementers understand these needs.  **Exclusion Criteria:**  Exclude statements that demonstrate a strong need for community-wide MDA and code to “Tension for Change.” Exclude statements related to engagement/IEC strategies and code as “Engaging.” | Community_member_needs |
| **Cosmopolitanism** | The degree to which the MOH or other organizations are networked with each other. | **Inclusion Criteria:**  Include descriptions of networks across Ministries, with non-Ministry partners, and cross-sectorial collaborations.  **Exclusion Criteria:**  Exclude and code to “Cosmopolitanism (LF)” if specific to linkages between STH and LF programmes. | Cosmopolitanism |
| **Cosmopolitanism (LF)** | The degree to which the MOH or organizations are networked with each other in the context of LF treatment. | **Inclusion Criteria:**  Include descriptions of networks across Ministries, with non-Ministry partners, and cross-sectorial collaborations  **Exclusion Criteria:**  Exclude and code to “Cosmopolitanism” if not specific to linkages between STH and LF programmes | Cosmopolitanism_LF |
| **External policy and setting** | External strategies to disseminate or encourage adoption of cMDA including policy and regulations, (governmental or other central entity), external mandates, recommendations and guidelines, pay-for-performance, collaboratives, and public or benchmark reporting. | **Inclusion Criteria**  Include statements that describe external performance measures from outside the country health system influencing effective MDA for STH delivery, such as WHO guidelines.  **Exclusion Criteria:**  None required | External_Policy |
| **DOMAIN: INNER SETTING**  *Features of implementing organization that may impact implementation* | | | |
| **Structural characteristics** | The social architecture, age, maturity, and size of an organization. | **Inclusion criteria:**  Include statements regarding changes needed to the health system to effectively deliver, sustain or scale community-wide MDA.  **Exclusion criteria:** Exclude statements regarding improved DeWorm3 study design or research infrastructure. | Structural_Characteristics |
| **Implementation climate** | The absorptive capacity for change, shared receptivity of involved individuals to an innovation, and the extent to which cMDA will be rewarded, supported, and expected within their organization. | **Inclusion Criteria**:  Include statements regarding the general level of receptivity to implementing community-wide MDA for STH *amongst a stakeholder group*, and reasons why.  **Exclusion criteria:**  Exclude statements about *an individual’s* level of receptivity to community-wide MDA and code to “Knowledge & beliefs about the intervention**”** | Impl_climate |
| **Implementation climate (LF)** | The absorptive capacity for change, shared receptivity of involved individuals to an innovation, and the extent to which use of that innovation will be rewarded, supported, and expected within their organization. | **Inclusion Criteria**:  Include any statements regarding the general level of receptivity to leveraging LF MDA platforms for community-wide MDA for STH *amongst a stakeholder group*, and reasons why.  **Exclusion criteria:**  Exclude statements about *an individual’s* level of receptivity to integrating LF programs and community-wide MDA and code to “Knowledge & beliefs about the intervention**”** | Impl_climate _LF |
| **Tension for change** | The degree to which stakeholders perceive the current situation as needing to change. | **Inclusion Criteria:**  Include statements demonstrating a strong need for community-wide MDA, or a strong indication that community-wide MDA is not needed.  **Exclusion criteria:** Exclude statements regarding specific needs of community-members that don’t necessarily indicate a strong need for the intervention generally and code to “Patient needs.” Exclude statements that demonstrate the innovation is better (or worse) than existing programs and code as “Relative Advantage.” | Tension_Change |
| **Learning climate** | A climate in which: 1. Leaders express their own fallibility and need for team members’ assistance and input; 2. Team members feel that they are essential, valued, and knowledgeable partners in the change process; 3. Individuals feel psychologically safe to try new methods; and 4. There is sufficient time and space for reflective thinking and evaluation. | **Inclusion Criteria:**  Include statements that support (or refute) the degree to which participating stakeholders or team members in their workplaces exhibit openness to the change process (cMDA activities).  **Exclusion Criteria:**  None required | Learning_Climate |
| **Compatibility** | The degree of tangible fit between meaning and values attached to the innovation by involved individuals, how those align with individuals’ own norms, values, and perceived risks and needs, and how the innovation fits with existing workflows and systems. | **Inclusion Criteria:**  Include statements that demonstrate the level of compatibility community-wide MDA has with government values and health system design. Include statements that the innovation did or did not need to be adapted as evidence of compatibility or lack of compatibility.  **Exclusion Criteria:**  Exclude or double code statements regarding the prioritization of community-wide MDA based on fit with organizational values as “Relative Priority”, (e.g., if an intervention is not prioritized because it is not compatible with MOH organizational values). | Compatibility |
| **Relative priority** | Individuals’ shared perception of the importance of implementation within the organization. | **Inclusion Criteria:**  Include statements that reflect the relative priority of community-wide MDA, for example, statements related to change fatigue in the MOH due to implementation of many other programs.  **Exclusion Criteria:**  Exclude or double code statements regarding the priority of the intervention based on compatibility with organizational values to “Compatibility.” | Relative_Priority |
| **Organizational incentives & rewards** | Extrinsic incentives such as goal-sharing, awards, performance reviews, promotions, and raises in salary, and less tangible incentives such as increased stature or respect. | **Inclusion Criteria**:  Include statements related to whether incentive systems are in place to encourage (or hinder) effective MDA delivery, for example rewards or disincentives for drug distributors engaging in the innovation.  **Exclusion Criteria:**  None required | Incentives |
| **Readiness for implementation** | Tangible and immediate indicators of organizational commitment to its decision to implement an innovation. | **Inclusion Criteria:**  Include statements regarding the general level of readiness for community-wide MDA.  **Exclusion Criteria:**  None required (will often require double coding with sub-constructs below) | Readiness_Implementation |
| **Readiness for implementation (health workforce)** | Tangible and immediate indicators of organizational commitment to its decision to implement an innovation. | **Inclusion Criteria:**  Include statements regarding the general level of readiness of the health workforce for community-wide MDA.  **Exclusion Criteria:**  Exclude or double code statements regarding resources that health workers need to be successful to “Available resources” | Readiness_ Implementation_workforce |
| **Available resources** | The level of resources organization dedicated for implementation and on-going operations including physical space and time. | **Inclusion Criteria:**  Include statements related to the presence or absence of resources specific to implementing community-wide MDA, or MDA generally  **Exclusion Criteria:**  Exclude statements related to the quality of materials and code to “Design Quality & Packaging.” Exclude statements related to resources needed for conducting the research components (e.g., time to complete research tasks, such as IRB applications). | Available_resources |
| **Leadership engagement** | Commitment, involvement, and accountability of leaders and managers with the implementation of the innovation. | **Inclusion Criteria:**  Include statements regarding the level of engagement of MOH or other relevant leadership.  **Exclusion Criteria:**  None required | Leadership_Engagement |
| **DOMAIN: CHARACTERISTICS OF INDIVIDUALS**  *Individuals involved in implementation* | | | |
| **Knowledge & beliefs about the intervention** | Individuals’ attitudes toward and value placed on cMDA as well as familiarity with facts, truths, and principles related to the innovation. | **Inclusion Criteria:**  Include statements reflecting an individual’s attitudes and beliefs about community-wide MDA, or community-based healthcare delivery.  **Exclusion Criteria:**  Exclude statements related to familiarity with evidence about community-wide MDA and code to “Evidence Strength & Quality” | Knowledge_Beliefs |
| **Self-efficacy** | Individual belief in their own capabilities to execute courses of action to achieve implementation goals. | **Inclusion criteria:**  Include statements regarding an individual’s beliefs about their own ability to effectively contribute to MDA for STH.  **Exclusion Criteria:**  None required | Self_Efficacy |
| ***DOMAIN: PROCESS***  *Strategies or tactics and presence of key intervention stakeholders that may impact implementation* | | | |
| **Planning** | The degree to which a plan or method for implementing cMDA is developed in advance, and the quality of those plans. | **Inclusion Criteria:**  Include statements that provide evidence of pre-cMDA assessments and planning, as well as refinements to the plan.  **Exclusion Criteria:**  None required | Planning |
| **Engaging: Opinion leaders** | Individuals in an organization that have formal or informal influence on the attitudes and beliefs of their colleagues with respect to implementing the innovation. | **Inclusion Criteria:**  Include statements related to engaging key leaders, and outcomes of engaging them in MDA planning or implementation.  **Exclusion Criteria:**  None required | Engaging_Leaders |
| **Engaging: Innovation participants** | Involving innovation participants through a combined strategy of social marketing, education, role modeling, training, and other similar activities. | **Inclusion Criteria:**  Include statements related to engagement strategies and outcomes for community members prior to or during MDA.  **Exclusion Criteria:**  Exclude or double code statements related to who participated in the decision process to implement the intervention to “Innovation Source”, as an indicator of internal or external source. | Engaging_Participants |
| **Executing** | Carrying out or accomplishing the implementation of cMDA according to plan. | **Inclusion criteria:** Any statements pertaining to meeting targets, activities meeting set goals. This includes perceptions of whether cMDA was implemented with fidelity to guidelines or original plans.  **Exclusion criteria:**  None required | Executing |
| **Reflecting/evaluative** | Quantitative and qualitative feedback about the progress and quality of implementation of cMDA, accompanied with regular personal and team debriefing about progress and experience. | **Inclusion criteria:** Any statements pertaining to evaluation of MDA as part of the implementation process (ex. quality of coverage survey implementation).  **Exclusion criteria:** Exclude statements that capture the reflecting and evaluating that participants may do during the FGD or interview, for example, related to the success of the implementation and code to [*Knowledge & Beliefs about the Intervention*](http://cfirwiki.net/wiki/index.php?title=Knowledge_%26_Beliefs_about_the_Intervention)*.* | Reflecting_Evaluative |
| **NON-CFIR CODES** | | | |
| **Gender (health workers)** | Gender and sex dynamics of government personnel and health workers that influence successful implementation of an intervention. | **Inclusion Criteria:**  Statements regarding how gender and sex of government personnel and health workers does or does not influence successful delivery of community-wide MDA  **Exclusion criteria:**  Double code to “Equity” for statements regarding how gender dynamics may or may not result in differences in healthcare access or MDA coverage by sex | Gender_Health_Worker |
| **Gender (community members)** | Gender and sex dynamics between community members and the environments that they live in, which influence successful implementation of an intervention. | **Inclusion Criteria:**  Statements regarding how gender and sex of community members does or does not influence successful delivery of community-wide MDA with high coverage  **Exclusion criteria:**  Double code to “Equity” for statements regarding how gender dynamics may or may not result in differences in healthcare access or MDA coverage by sex | Gender_Community |
| **Scalability** | The likelihood that an innovation can be disseminated and spread to other geographic or implementation organizations. | **Inclusion Criteria:**  Statements regarding the likelihood or unlikelihood that community-wide MDA for STH will be incorporated as policy or practice across wider geographic areas than DeWorm3.  **Exclusion criteria:**  Exclude statements that demonstrate the level of compatibility community-wide MDA has with government values and health system design and code as “Compatibility”. | Scalability |
| **Equity** | The condition in which an innovation or intervention is accessible, of high quality, and taken-up equitable across populations and sub-populations. | **Inclusion Criteria:**  Statements regarding the ability or inability for STH MDA to reach populations and sub-populations with similar quality or coverage.  **Exclusion criteria:**  None required | Equity |
| **Donor relationships** | Involving relationships between donors, researchers, and implementers and the way in which those relationships influence innovation or implementation. | **Inclusion Criteria:**  Statements regarding the role or influence (or lack thereof) of donor relationships on STH elimination policy and activities.  **Exclusion criteria:**  None required | Donor_ relationships |
| **STH Knowledge** | Individuals’ beliefs and knowledge of STH, as well as familiarity with facts, truths, and principles related to STH. | **Inclusion Criteria:**  Include statements reflecting an individual’s beliefs and knowledge of STH, STH transmission or behaviors associated with STH.  **Exclusion Criteria:**  Exclude statements related to familiarity with evidence about community-wide MDA and code to “Knowledge_Beliefs” | STH_knowledge |
| **Community health program** | Description of existing community health programs | **Inclusion Criteria:**  Include statements regarding other community health programs being implemented in target communities. For example, if people mention malaria programs without linking them to the cMDA programs or expressing attitudes and beliefs about community health programs in general.  **Exclusion Criteria:**  Exclude statements that compare other community health programs to cMDA and code to the appropriate CFIR construct such as “Relative Advantage” or statements that express attitudes, beliefs, and familiarity with facts about a community health program and code to “Knowledge_Beliefs”. | Community_program |
| **LF Experience** | Individuals’ beliefs and attitudes towards and knowledge about lymphatic filariasis programs. | **Inclusion Criteria:**  Statements reflecting an individual’s beliefs and attitudes towards LF programs, without linking them to cMDA programs in any way.  **Exclusion Criteria:**  Exclude statements comparing the LF program to the cMDA program and code to “Relative Advantage”. Exclude statements about how LF platforms were leveraged in cMDA implementation and code to appropriate CFIR LF contruct (e.g., “Design_Packaging_LF”, “Impl_climate _LF”) | LF_experience |
| **Trachoma Experience** | Individuals’ beliefs and attitudes towards and knowledge about trachoma programs. | **Inclusion Criteria:**  Statements reflecting an individual’s beliefs and attitudes towards trachoma programs, without linking them to cMDA programs in any way.  **Exclusion Criteria:**  Exclude statements comparing the Trachoma program to the cMDA program and code to “Relative Advantage”. | Trachoma_experience |
| **Trial Conduct** | Individuals’ understanding of and description of elements of the DeWorm3 trial including stool sample collection and household surveys. | **Inclusion Criteria:**  Statements in which aspects of the DeWorm3 trial are discussed (e.g., collection of stool samples) that may not be relevant to cMDA programs operating outside of a trial format. For example, discussions of DeWorm3 identification cards or prevalence surveys.  **Exclusion Criteria:**  None required | Trial_conduct |
| **Recommendations** | Feedback on how the cMDA program can be improved. | **Inclusion Criteria:**  Statements regarding recommendations for improvement of cMDA programs. This code will always be double coded with a CFIR construct such as “Design quality and packaging”, “Engaging participants”, etc.  **Exclusion Criteria:**  Statements regarding observed or potential challenges changing cMDA to accommodate recommendations should be coded as “Adaptability”. | Recommendation |
