# Supplementary material for: “Our desire is to make this village intestinal worm free”: Identifying determinants of high coverage of community-wide mass drug administration for soil transmitted helminths in Benin, India, and Malawi
Source: PLoS Negl Trop Dis. 2024 Feb 6;18(2):e0011819. doi: 10.1371/journal.pntd.0011819 (PMC10846705; doi:10.1371/journal.pntd.0011819)
Supplement: S3 Appendix — (DOCX) [file pntd.0011819.s003.docx]

**S3 COREQ Table**

| **No** | **Item** | **Guide questions/description** | **Response/Page number referenced** |
| --- | --- | --- | --- |
| **Domain 1: Research team and reflexivity** | | | |
| Personal Characteristics | | | |
| 1. | Interviewer/  facilitator | Which author/s conducted the interview or focus group? | PN, CMC, AT, JJ, CIT, FC |
| 2. | Credentials | What were the researcher's credentials? E.g. PhD, MD | MS (MSc)*, AR (MPH)*, JLW (MD), EP (MPH), HL (MSc), PN (MSc), CMC (MPH), AT (MSW), JJ (BSc), MSW), EAB (PhD), CIT (MSc), FC (MSc), EA (MD), KK (MD), SSRA (MD, PhD), MI (PhD), KA (MD), ARM (PhD) |
| 3. | Occupation | What was their occupation at the time of the study? | MS (DeWorm3 Social Scientist), AR (Implementation Science (IS) Data Analyst), JLW (DeWorm3 study PI; Professor University of Washington), EP (Data Manager), HL (DeWorm3 Clinical Trials Operations Coordinator, LSHTM),PN (DeWorm3 IS Assistant), CMC (DeWorm3 IS Assistant), AT and JJ (DeWorm3 Social Scientists), EAB (Research Assistant), FC, CIT (DeWorm3 IS Assistants), EA (DeWorm3 site deputy coordinator), KK (DeWorm3 site PI), SSRA (DeWorm3 India site PI and Professor, The Christian Medical College, Vellore), MI (DeWorm3 site PI; Professor of Parasitology at University of Abomey-Calavi), KA (DeWorm3 Implementation Science Coordinator, India); ARM (DeWorm3 Implementation Science PI, Assistant Professor University of Washington) |
| 4. | Gender | Was the researcher male or female? | Both male and female researchers were involved. However, this is not relevant in the context of this study as would not have affected the conduct or analysis of FGDs on facilitators and barriers to achieving high coverage in a cMDA for STH program. |
| 5. | Experience and training | What experience or training did the researcher have? | Page 3 (methods): FGD facilitators were trained on data collection best practices using site adapted FGD guides. |
| Relationship with Participants | | | |
| 6. | Relationship established | Was a relationship established prior to study commencement? | Participants were selected from DeWorm3 census lists. No prior relationship established with participants prior to study commencement. |
| 7. | Participant knowledge of the interviewer | What did the participants know about the researcher? e.g. personal goals, reasons for doing the research | Participants knew the purpose of the DeWorm3 study. |
| 8. | Interviewer characteristics | What characteristics were reported about the interviewer/facilitator? e.g. Bias, assumptions, reasons, and interests in the research topic | FGDs were conducted by experienced qualitative researchers specifically trained on DeWorm3 question guides and process of conducting FGDs. |
| **Domain 2: study design** | | | |
| Theoretical framework | | | |
| 9. | Methodological orientation and Theory | What methodological orientation was stated to underpin the study? e.g. grounded theory, discourse analysis, ethnography, phenomenology, content analysis | Page 2 (methods section): The Consolidated Framework for Implementation Research (CFIR) is a meta-theoretical determinants framework of 39 constructs divided into five thematic domains: inner setting, outer setting, process, individuals involved, and intervention characteristics. The CFIR was selected to guide the investigation of the multi-level factors influencing coverage across clusters. |
| Participant selection | | | |
| 10. | Sampling | How were participants selected? e.g. purposive, convenience, consecutive, snowball | Page 2 (methods): In Benin and India, men, women, and children were randomly selected from the population census. In Malawi, one village was randomly selected, and the participants were purposively selected along with village leader. The local leaders were purposively selected from each cluster. In India, participation was restricted to individuals who lived within 5 kilometers of the interview site given significant access challenges. |
| 11. | Method of approach | How were participants approached? e.g. face-to-face, telephone, mail, email | Page 2 (methods): The participants in India were contacted in-person; participants in Benin were contacted in-person (or telephonically when needed). The participants in Malawi were invited through the village chief. The team ensured no more than one individual per household was selected. |
| 12. | Sample size | How many participants were in the study? | Page 2 (methods): About 5-10 individuals participated in each FGD. A total of 48 FGDs were conducted. |
| 13. | Non-participant | How many people refused to participate or dropped out? Reasons? | There were no refusals in Benin and India. In Malawi, 15 out of 159 did not come for interview after accepting the invitation to participant. |
| Setting | | | |
| 14. | Setting of data collection | Where was the data collected? e.g. home, clinic, workplace | Page 3 (methods): FGDs were conducted in private locations with a facilitator and a notetaker. |
| 15. | Presence of non-participants | Was anyone else present besides the participants and researchers? | Not applicable, no other groups were present during conduct of focus group discussions. |
| 16 | Description of sample | What are the important characteristics of the sample? e.g. demographic data, date | Page 2 (methods): Table 1 Overview of study sites |
| Data collection | | | |
| 17. | Interview guide | Were questions, prompts, guides provided by the authors? Was it pilot tested? | Page 2-3 (methods): Thirty-two CFIR constructs across all five domains and seven non-CFIR constructs hypothesized to influence cMDA were identified a priori to develop semi-structured question guides [see Additional file 1]. A common guide was used for local leaders, adults, and children. which were adapted and translated within each site for cultural appropriateness and iteratively adapted as necessary. |
| 18. | Repeat interviews | Were repeat interviews carried out? If yes, how many? | Not applicable – repeat interviews were not conducted. |
| 19. | Audio/visual recording | Did the research use audio or visual recording to collect the data? | Page 3 (methods): Following consenting and assenting procedures FGDs were audio recorded. Audio files were transcribed in the local language and translated into English. Complete double checks in India and Malawi and random spot-checks in Benin were performed for quality assurance at each stage. |
| 20. | Field notes | Were field notes made during and/or after the interview or focus group? | Page 3 (methods): Field notes were made during the FGDs by a trained notetaker. |
| 21. | Duration | What was the duration of the interviews or focus group? | Duration of focus group discussions were between 25 and 120 minutes. |
| 22. | Data saturation | Was data saturation discussed? | Determination of data saturation was not an objective of this research study. Data saturation was reached within sites; however, heterogeneity was present across sites. |
| 23. | Transcripts returned | Were transcripts returned to participants for comment and/or correction? | Transcripts were not returned to study participants. |
| **Domain 3: analysis and findings** | | | |
| Data analysis | | | |
| 24. | Number of data coders | How many data coders coded the data? | Page 3 (methods): Coders were based in each DeWorm3 site with one coder at the University of Washington (UW), Seattle (total of five coders, two based in Malawi). |
| 25. | Description of the coding tree | Did authors provide a description of the coding tree? | A coding tree was not made for this study. |
| 26. | Derivation of themes | Were themes identified in advance or derived from the data? | Page 3 (methods): A mix of deductive and inductive coding was used, with an initial codebook developed based on CFIR constructs with contextualized definitions [see Additional file 2]. During data analysis, inductive codes were needed to describe nuances in community member behavior. |
| 27. | Software | What software, if applicable, was used to manage the data? | Atlas.ti (8.4.5) |
| 28. | Participant checking | Did participants provide feedback on the findings? | Participants did not provide feedback on the findings. |
| Reporting | | | |
| 29. | Quotations presented | Were participant quotations presented to illustrate the themes / findings? Was each quotation identified? e.g. participant number | Pages 4-19 (Results section): Quotations are presented throughout the text alongside interpretations. |
| 30. | Data and findings consistent | Was there consistency between the data presented and the findings? | Pages 4-19 (Results section) and Page 19-21 (Discussion section): Quotations are presented throughout the text in the Results section alongside interpretations. The meaning of quotations was further explored in the Discussion section. |
| 31. | Clarity of major themes | Were major themes clearly presented in the findings? | Pages 4-19 (Results section): Major themes are presented and organized as key facilitators, barriers, and if the qualitative and quantitative data converge that affect implementation of cMDA with high coverage. |
| 32. | Clarity of minor themes | Is there a description of diverse cases or discussion of minor themes? | Pages 4-19 (Results section): Minor themes are discussed in more detail through quotations and interpretations under major theme headings. |
