# Supplementary material for: “Our desire is to make this village intestinal worm free”: Identifying determinants of high coverage of community-wide mass drug administration for soil transmitted helminths in Benin, India, and Malawi
Source: PLoS Negl Trop Dis. 2024 Feb 6;18(2):e0011819. doi: 10.1371/journal.pntd.0011819 (PMC10846705; doi:10.1371/journal.pntd.0011819)
Supplement: S4 Appendix — (DOCX) [file pntd.0011819.s004.docx]

**S4 CFIR construct rating rules***

| Rating | Criteria |
| --- | --- |
| -2 | The construct has a negative influence on community wide MDA (cMDA) coverage. It has an impeding influence on MDA implementation processes, and/or an impeding influence on MDA implementation efforts. There is a strong sentiment in the FGD that shows how a construct manifests itself in a negative way. |
| -1 | The construct is a negative influence on cMDA coverage. It has an impeding influence in MDA implementation processes, and/or an impeding influence in cMDA implementation efforts. Respondents make general statements about the construct manifesting in a negative way but without concrete examples: (1) the construct is mentioned only in passing or at a high level without examples or evidence of actual, concrete descriptions of how that construct manifests; (2) there is a mixed effect of different aspects of the construct but with a general overall negative effect; (3) there is sufficient information to make an indirect inference about the generally negative influence; and/or (4) judged as weakly negative by the absence of the construct. |
| 0 | A construct has neutral influence on cMDA coverage if: (1) it appears to have neutral effect (purely descriptive) or is only mentioned generically without valence; (2) there is no evidence of positive or negative influence; (3) credible or reliable respondents contradict each other. |
| +1 | The construct has a positive influence for cMDA coverage. It has a facilitating influence on MDA implementation processes, and/or a facilitating influence in cMDA implementation efforts. Respondents make general statements about the construct manifesting in a positive way but without concrete examples: (1) the construct is mentioned only in passing or at a high level without examples or evidence of actual, concrete descriptions of how that construct manifests; (2) there is a mixed effect of different aspects of the construct but with a general overall positive effect; and/or (3) there is sufficient information to make an indirect inference about the generally positive influence. |
| +2 | The construct has a positive influence on cMDA coverage. It has a facilitating influence on MDA implementation processes, and/or a facilitating influence on MDA implementation efforts. The majority of respondents describe explicit examples of how the key (or all) aspects of a construct manifests in a positive way. |
| Not Present | Respondent(s) were not asked about the presence or influence of the construct or, if they were asked about a construct, their responses did not correspond to the intended construct and were instead coded to another construct. Respondent(s)’ lack of knowledge about a construct does not necessarily indicate missing data and may instead indicate the absence of the construct. |

*Adapted from: Soi C, Gimbel S, Chilundo B, Muchanga V, Matsinhe L, Sherr K. Human papillomavirus vaccine delivery in Mozambique: identification of implementation performance drivers using the Consolidated Framework for Implementation Research (CFIR). Implementation Science. 2018 Dec;13(1):1-2.
