# Supplementary material for: “Our desire is to make this village intestinal worm free”: Identifying determinants of high coverage of community-wide mass drug administration for soil transmitted helminths in Benin, India, and Malawi
Source: PLoS Negl Trop Dis. 2024 Feb 6;18(2):e0011819. doi: 10.1371/journal.pntd.0011819 (PMC10846705; doi:10.1371/journal.pntd.0011819)
Supplement: S5 Appendix — (DOCX) [file pntd.0011819.s005.docx]

**S5 Quantitative full tables**

|  |
| --- |

|  | | **Benin High Coverage** | | | **Benin Low Coverage** | | | **India High Coverage** | | | **India Low Coverage** | | **Malawi High Coverage** | | **Malawi Low Coverage** |  |  |  |
| --- | --- | --- | --- | --- | --- | --- | --- | --- | --- | --- | --- | --- | --- | --- | --- | --- | --- | --- |
|  | | **%(n)** | | | **%(n)** | | | **%(n)** | | | **%(n)** | | **%(n)** | | **%(n)** |  |  |  |
|  | |  | | |  | | |  | | |  | |  | |  |  |  |  |
| **Coverage Measures** | | **N=1046** | | | | **N=1235** | **N=1135** | | | **N=1122** | | **N=1189** | | **N=1243** | |  |  |  |
|  |  |  | | |  | | |  | | |  | |  | |  |  |  |  |
| *Treatment Recall* | | | | | | | | | | | | | | | |  |  |  |
| Remember receiving albendazole |  | 98.2(1027) | | | 90.9(1123) | | | 90.6(1028) | | | 96.4(1082) | | 90.4(1075) | | 82.9(1030) |  |  |  |
|  | | | | | | | | | | | | | | | |  |  |  |
| *Among Those Who Remember Treatment* | | | | | | | | | | | | | | | |  |  |  |
| Males |  | 47.7(490) | | | 49.5(556) | | | 48.2(495) | | | 48.3(523) | | 45.1(485) | | 42.4(437) |  |  |  |
|  | | | | | | | | | | | | | | | |  |  |  |
| Females |  | 52.3(537) | | | 50.5(567) | | | 51.8(533) | | | 51.7(559) | | 54.9(590) | | 57.5(592) |  |  |  |
|  | | | | | | | | | | | | | | | |  |  |  |
| Aware of MDA prior to distribution, total* |  | 77.5(796) | | | 86.2(968) | | | 49.5(509) | | | 54.1(585) | | 37.9(407) | | 72.8(750) |  |  |  |
|  | Aware from, family/friend | 55.0(438) | | | 81.2(786) | | | 42.8(218) | | | 55.6(325) | | 41.8(170) | | 24.4(183) |  |  |  |
|  | Aware from, health staff | 0.6(5) | | | 1.2(12) | | | 0.2(1) | | | 6.0(35) | | 11.8(48) | | 22.9(172) |  |  |  |
|  | Aware from, CDD/CHW/teacher^1^ | 60.6(482) | | | 17.5(169) | | | 11.2(57) | | | 9.6(56) | | 45.7(186) | | 40.8(306) |  |  |  |
|  | Aware from, community leader | 4.5(36) | | | 6.3(61) | | | 0.2(1) | | | 0.2(1) | | 7.4(30) | | 21.1(158) |  |  |  |
|  | Aware from, brochures/flyers | 1.0(8) | | | 0.0(0) | | | 1.2(6) | | | 2.2(13) | | 1.0(4) | | 0.8(6) |  |  |  |
|  | Aware from, posters | 0.0(0) | | | 0.1(1) | | | 18.5(94) | | | 20.0(117) | | 0.0(0) | | 0.0(0) |  |  |  |
|  | Aware from, banners | 0.0(0) | | | 0.3(3) | | | 25.9(132) | | | 33.2(194) | | 0.0(0) | | 0.0(0) |  |  |  |
|  | Aware from, radio | 0.0(10.0) | | | 0.2(153.0) | | | 0.0(2.0) | | | 0.0(15.0) | | 0.0(0.0) | | 0.0(2.0) |  |  |  |
|  | Aware from, TV | 0.0(0) | | | 0.0(0) | | | 0.0(0) | | | 0.5(3) | | 0.0(0) | | 0.0(0) |  |  |  |
|  | Aware from, social media | 0.1(1) | | | 0.0(0) | | | 1.4(7) | | | 0.2(1) | | 0.0(0) | | 0.0(0) |  |  |  |
|  | Aware from, community theater |  | | |  | | | 37.3(190) | | | 9.7(57) | | 21.9(89) | | 26.8(201) |  |  |  |
|  | Aware from, Gram Sabha^2^ |  | | |  | | | 7.1(36) | | | 1.4(8) | |  | |  |  |  |  |
|  | Aware from, PA System |  | | |  | | |  | | |  | | 0.2(1) | | 1.6(12) |  |  |  |
|  | | | | | | | | | | | | | | | |  |  |  |
| Accepted treatment |  | 99.7(1024) | | | 99.6(1118) | | | 97.9(1006) | | | 99.4(1076) | | 98.7(1061) | | 100.0(1030) |  |  |  |
|  | Reported side-effects | 2.7(28) | | | 2.3(26) | | | 1.2(12) | | | 2.4(26) | | 8.0(85) | | 3.2(33) |  |  |  |
|  | | | | | | | | | | | | | | | |  |  |  |
| Did not accept treatment |  | 0.3(3) | | | 0.4(5) | | | 1.7(17) | | | 0.4(4) | | 1.2(13) | | 0.0(0) |  |  |  |
|  | Pregnant | 33.3(1) | | | 0.0(0) | | | 17.6(3) | | | 0.0(0) | | 15.4(2) | |  |  |  |  |
|  | Breastfeeding | 0.0(0) | | | 0.0(0) | | | 0.0(0) | | | 0.0(0) | | 0.0(0) | |  |  |  |  |
|  | Ill | 33.3(1) | | | 0.0(0) | | | 5.9(1) | | | 25.0(1) | | 0.0(0) | |  |  |  |  |
|  | Too old | 0.0(0) | | | 0.0(0) | | | 5.9(1) | | | 0.0(0) | | 0.0(0) | |  |  |  |  |
|  | Less than 1 years old | 0.0(0) | | | 0.0(0) | | | 0.0(0) | | | 25.0(1) | | 38.5(5) | |  |  |  |  |
|  | Afraid of side effects | 0.0(0) | | | 20.0(1) | | | 5.9(1) | | | 0.0(0) | | 0.0(0) | |  |  |  |  |
|  | Believe they are not at risk for this disease | 0.0(0) | | | 0.0(0) | | | 5.9(1) | | | 0.0(0) | | 0.0(0) | |  |  |  |  |
|  | Taking other medications | 33.3(1) | | | 0.0(0) | | | 17.6(3) | | | 0.0(0) | | 7.7(1) | |  |  |  |  |
|  | Believe that medicine does not work | 0.0(0) | | | 0.0(0) | | | 0.0(0) | | | 0.0(0) | | 0.0(0) | |  |  |  |  |
|  | Don't know/cannot recall | 0.0(0) | | | 0.0(0) | | | 35.3(6) | | | 25.0(1) | | 0.0(0) | |  |  |  |  |
|  | Refused to answer | 0.0(0) | | | 60.0(3) | | | 5.9(1) | | | 25.0(1) | | 30.8(4) | |  |  |  |  |
|  | Other | 0.0(0) | | | 20.0(1) | | | 0.0(0) | | | 0.0(0) | | 7.7(1) | |  |  |  |  |
|  | | | | | | | | | | | | | | | |  |  |  |
| Treated at home/in community^3^ |  | 94.7(973) | | | 94.0(1056) | | | 80.3(825) | | | 81.9(886) | | 90.7(975) | | 93.4(962) |  |  |  |
|  | Perceived neighbors took MDA | 96.7(941) | | | 84.5(892) | | | 78.9(651) | | | 63.9(566) | | 89.4(872) | | 90.1(867) |  |  |  |
|  | | | | | | | | | | | | | | | |  |  |  |
| *MDA Time/Preferences^4^* | | | | | | | | | | | | | | | |  |  |  |
| Preferred MDA distribution method |  |  | | |  | | |  | | |  | |  | |  |  |  |  |
|  | Door-to-door | 96.7(828) | | | 97.5(921) | | | 88.5(670) | | | 83.9(637) | | 74.9(555) | | 79.8(583) |  |  |  |
|  | Distribution at schools | 0.0(0) | | | 1.0(9) | | | 0.0(0) | | | 0.5(4) | | 1.8(13) | | 0.7(5) |  |  |  |
|  | Distribution at central location | 0.0(0) | | | 0.2(2) | | | 0.1(1) | | | 0.9(7) | | 0.9(7) | | 2.5(18) |  |  |  |
|  | Other distribution method | 2.2(19) | | | 0.0(0) | | | 0.0(0) | | | 0.0(0) | | 0.3(2) | | 0.0(0) |  |  |  |
|  | Don't know/refused/missing | 1.1(9) | | | 1.4(13) | | | 11.4(86) | | | 14.6(111) | | 22.1(164) | | 17.1(125) |  |  |  |
|  | | | | | | | | | | | | | | | |  |  |  |
| Liked which aspect(s) of MDA?* |  |  | | |  | | |  | | |  | |  | |  |  |  |  |
|  | House-house treatment | 94.6(810) | | | 69.3(655) | | | 86.4(654) | | | 83.3(632) | | 43.3(321) | | 57.5(420) |  |  |  |
|  | Trusted distributors | 24.4(209) | | | 23.0(217) | | | 2.2(17) | | | 13.4(102) | | 8.4(62) | | 17.9(131) |  |  |  |
|  | Short wait time | 22.0(188) | | | 5.9(56) | | | 0.1(1) | | | 11.9(90) | | 6.9(51) | | 22.3(163) |  |  |  |
|  | Free drugs | 24.9(213) | | | 21.0(198) | | | 2.6(20) | | | 4.0(30) | | 21.3(158) | | 48.6(355) |  |  |  |
|  | Liked no specific aspect | 1.3(11) | | | 12.5(118) | | | 0.0(0) | | | 0.8(6) | | 7.3(54) | | 2.3(17) |  |  |  |
|  | | | | | | | | | | | | | | | |  |  |  |
| Disliked which aspect(s) of MDA?* |  |  | | |  | | |  | | |  | |  | |  |  |  |  |
|  | Inconvenient Time | 0.1(1) | | | 10.5(99) | | | 30.5(231) | | | 44.9(341) | | 15.5(115) | | 5.5(40) |  |  |  |
|  | Drugs not available | 0.0(0) | | | 0.1(1) | | | 0.0(0) | | | 0.1(1) | | 1.1(8) | | 0.4(3) |  |  |  |
|  | Unfriendly distributor | 0.2(2) | | | 1.0(9) | | | 0.0(0) | | | 1.2(9) | | 1.1(8) | | 1.5(11) |  |  |  |
|  | Took too much time | 0.0(0) | | | 5.7(54) | | | 8.9(67) | | | 5.8(44) | | 10.0(74) | | 5.2(38) |  |  |  |
|  | Adverse drug reaction | 0.5(4) | | | 1.3(12) | | | 0.0(0) | | | 0.3(2) | | 0.7(5) | | 0.0(0) |  |  |  |
|  | No treatment for other diseases | 36.9(316) | | | 18.1(171) | | | 30.5(231) | | | 8.6(65) | | 2.4(18) | | 10.1(74) |  |  |  |
|  | Disliked no specific aspect | 58.8(503) | | | 66.7(630) | | | 4.2(32) | | | 18.8(143) | | 46.3(343) | | 56.4(412) |  |  |  |
|  |  |  | | |  | | |  | | |  | |  | |  |  |  |  |
| **Individual Level Drug Distribution Measures** | | **N=14500** | | | | **N=12893** | **N=24638** | | | **N=22286** | | **N=20625** | | **N=17233** | |  |  |  |
| *Treatment* | | | | | | | | | | | | | | | |  |  |  |
| Treatment Status |  |  | | |  | | |  | | |  | |  | |  |  |  |  |
|  | Directly observed therapy | 86.3 (12512) | | | 69.3 (8934) | | | 72.5 (17863) | | | 70.9 (15802) | | 64.0 (13190) | | 60.9 (10491) |  |  |  |
|  | Individual was recently treated | 5.6 (819) | | | 6.1 (782) | | | 17.4 (4284) | | | 16.5 (3678) | | 8.2 (1690) | | 6.0 (1037) |  |  |  |
|  | Not treated | 7.8 (1127) | | | 21.0 (2710) | | | 6.4 (1572) | | | 9.5 (2116) | | 18.7 (3852) | | 29.1 (5016) |  |  |  |
|  | Tablet was left at house | 0.2 (25) | | |  | | | 2.6 (649) | | | 2.0 (449) | | 0.2 (32) | | 1.0 (164) |  |  |  |
|  | Tablet was left with participant | 0.1 (17) | | | 3.6 (467) | | | 1.1 (270) | | | 1.1 (241) | | 9.0 (1861) | | 3.0 (525) |  |  |  |
|  | | | | | | | | | | | | | | | |  |  |  |
| *Gender Community* | | | | | | | | | | | | | | | |  |  |  |
| Eligible Adults^5^ |  | 60.5(8772) | | | 59.9(7728) | | | 77.9(19195) | | | 80.9(18023) | | 52.3(10796) | | 51.8(8922) |  |  |  |
|  | Eligible Adult Females | 32.6(4734) | | | 32.5(4194) | | | 39.9(9837) | | | 41.0(9137) | | 29.1(6003) | | 28.7(4952) |  |  |  |
|  | Eligible Adult Males | 27.8(4038) | | | 27.4(3534) | | | 38.0(9358) | | | 39.9(8886) | | 23.2(4793) | | 23.0(3970) |  |  |  |
|  | | | | | | | | | | | | | | | |  |  |  |
| Treatment Status Among Eligible Adult Males |  |  | | |  | | |  | | |  | |  | |  |  |  |  |
|  | Directly observed therapy | 90.0(3636) | | | 71.3(2518) | | | 84.1(7873) | | | 82.0(7284) | | 54.5(2614) | | 50.5(2003) |  |  |  |
|  | Individual was recently treated | 2.5(101) | | | 1.8(63) | | | 4.1(387) | | | 3.2(287) | | 3.1(150) | | 1.9(74) |  |  |  |
|  | Not treated | 7.2(289) | | | 22.6(799) | | | 6.0(557) | | | 10.2(908) | | 26.1(1249) | | 38.2(1515) |  |  |  |
|  | Tablet was left at house | 0.2(8) | | |  | | | 4.1(384) | | | 2.8(251) | | 0.4(20) | | 2.3(92) |  |  |  |
|  | Tablet was left with participant | 0.1(4) | | | 4.4(154) | | | 1.7(157) | | | 1.8(156) | | 15.9(760) | | 7.2(286) |  |  |  |
|  | | | | | | | | | | | | | | | |  |  |  |
| Treatment Status Among Eligible Adult Females |  |  | | |  | | |  | | |  | |  | |  |  |  |  |
|  | Directly observed therapy | 91.2(4319) | | | 75.1(3150) | | | 86.9(8549) | | | 85.3(7792) | | 77.7(4664) | | 74.6(3696) |  |  |  |
|  | Individual was recently treated | 1.4(67) | | | 2.0(84) | | | 4.2(409) | | | 4.3(395) | | 1.8(106) | | 1.8(90) |  |  |  |
|  | Not treated | 7.0(332) | | | 19.3(809) | | | 5.9(580) | | | 7.8(714) | | 10.8(651) | | 20.5(1015) |  |  |  |
|  | Tablet was left at house | 0.2(9) | | |  | | | 2.1(206) | | | 1.8(167) | | 0.1(5) | | 0.5(24) |  |  |  |
|  | Tablet was left with participant | 0.1(7) | | | 3.6(151) | | | 0.9(93) | | | 0.8(69) | | 9.6(577) | | 2.6(127) |  |  |  |
|  | | | | | | | | | | | | | | | |  |  |  |
| Reason for No Treatment in Eligible Adult Males |  |  | | |  | | |  | | |  | |  | |  |  |  |  |
|  | House not located | 0.3(1) | | | 8.5(68) | | | 1.3(7) | | | 0.8(7) | | 3.7(46) | | 13.8(209) |  |  |  |
|  | Household members not present | 6.2(18) | | | 27.3(218) | | | 11.7(65) | | | 11.9(108) | | 9.0(113) | | 14.9(225) |  |  |  |
|  | Intoxicated | 0.3(1) | | |  | | |  | | | 0.6(5) | | 0.1(1) | |  |  |  |  |
|  | Missing | 0.3(1) | | |  | | |  | | |  | | 0.2(2) | | 0.1(1) |  |  |  |
|  | Not present for treatment | 91.0(263) | | | 40.1(320) | | | 75.4(420) | | | 81.1(736) | | 73.7(920) | | 53.3(807) |  |  |  |
|  | Refused Treatment | 0.7(2) | | | 5.5(44) | | | 3.1(17) | | | 0.9(8) | | 6.2(78) | | 9.2(140) |  |  |  |
|  | Seriously ill | 1.0(3) | | | 0.4(3) | | | 6.6(37) | | | 3.4(31) | | 0.8(10) | | 0.7(11) |  |  |  |
|  | House visit was not attempted |  | | | 5.5(44) | | | 1.1(6) | | | 0.4(4) | | 2.8(35) | | 1.3(19) |  |  |  |
|  | Ineligible |  | | | 0.3(2) | | |  | | |  | |  | |  |  |  |  |
|  | should not be here |  | | | 12.5(100) | | | 0.9(5) | | | 1.0(9) | | 3.5(44) | | 6.8(103) |  |  |  |
|  | | | | | | | | | | | | | | | |  |  |  |
| Reason for No Treatment in Eligible Adult Females |  |  | | |  | | |  | | |  | |  | |  |  |  |  |
|  | House not located | 0.3(1) | | | 8.2(66) | | | 1.9(11) | | | 1.0(7) | | 9.5(62) | | 22.5(228) |  |  |  |
|  | Household members not present | 9.9(33) | | | 31.0(251) | | | 13.3(77) | | | 17.9(128) | | 20.0(130) | | 22.9(232) |  |  |  |
|  | Intoxicated |  | | | 0.1(1) | | |  | | |  | |  | |  |  |  |  |
|  | Missing | 0.3(1) | | | 0.2(2) | | |  | | |  | | 0.2(1) | | 0.2(2) |  |  |  |
|  | Not present for treatment | 84.9(282) | | | 36.7(297) | | | 65.7(381) | | | 73.2(523) | | 50.2(327) | | 38.3(389) |  |  |  |
|  | Refused Treatment | 0.9(3) | | | 3.6(29) | | | 3.8(22) | | | 1.3(9) | | 3.5(23) | | 2.8(28) |  |  |  |
|  | Seriously ill | 2.4(8) | | | 0.6(5) | | | 12.1(70) | | | 5.2(37) | | 1.7(11) | | 0.9(9) |  |  |  |
|  | House visit was not attempted |  | | | 6.9(56) | | | 1.2(7) | | | 0.1(1) | | 7.5(49) | | 1.7(17) |  |  |  |
|  | Ineligible | 0.3(1) | | | 0.2(2) | | |  | | |  | |  | |  |  |  |  |
|  | should not be here | 0.9(3) | | | 12.4(100) | | | 2.1(12) | | | 1.3(9) | | 7.4(48) | | 10.8(110) |  |  |  |
|  | | | | | | | | | | | | | | | |  |  |  |
| Time to Treatment Among All Treated Eligible Adult^6^ |  |  | | |  | | |  | | |  | |  | |  |  |  |  |
|  | Treated in 1st. visit | 92.7(7553) | | | 94.7(5793) | | | 86.8(15678) | | | 88.2(14460) | | 88.7(7894) | | 82.9(5298) |  |  |  |
|  | Treated in 2nd. visit | 6.9(565) | | | 5.2(317) | | | 7.7(1387) | | | 9.7(1597) | | 10.3(915) | | 14.1(903) |  |  |  |
|  | Treated in 3rd. visit | 0.4(29) | | | 0.2(10) | | | 3.6(652) | | | 1.7(284) | | 0.9(81) | | 2.6(165) |  |  |  |
|  | Treated in 4th. visit | 0.0(3) | | |  | | | 1.5(270) | | | 0.3(52) | | 0.1(6) | | 0.3(22) |  |  |  |
|  | Treated in 5th. visit or later |  | | |  | | | 0.4(67) | | | 0.0(8) | |  | | 0.1(4) |  |  |  |
|  | Unknown | 0.0(1) | | |  | | | 0.0(4) | | |  | |  | |  |  |  |  |
|  | | | | | | | | | | | | | | | |  |  |  |
| Time to Treatment Among Treated Eligible Adult Males^6^ |  |  | | |  | | |  | | |  | |  | |  |  |  |  |
|  | Treated in 1st. visit | 91.1(3414) | | | 93.2(2548) | | | 83.1(7310) | | | 84.5(6744) | | 80.1(2838) | | 71.1(1746) |  |  |  |
|  | Treated in 2nd. visit | 8.6(321) | | | 6.6(181) | | | 9.9(871) | | | 12.6(1009) | | 18.3(649) | | 23.1(567) |  |  |  |
|  | Treated in 3rd. visit | 0.3(13) | | | 0.2(6) | | | 4.8(420) | | | 2.3(185) | | 1.5(52) | | 5.1(126) |  |  |  |
|  | Treated in 4th. visit | 0.0(1) | | |  | | | 1.8(161) | | | 0.4(35) | | 0.1(5) | | 0.5(13) |  |  |  |
|  | Treated in 5th. visit or later |  | | |  | | | 0.4(38) | | | 0.1(5) | |  | | 0.1(3) |  |  |  |
|  | Unknown |  | | |  | | | 0.0(1) | | |  | |  | |  |  |  |  |
|  | | | | | | | | | | | | | | | |  |  |  |
| Time to Treatment Among Treated Eligible Adult Females^6^ |  |  | | |  | | |  | | |  | |  | |  |  |  |  |
|  | Treated in 1st. visit | 94.0(4139) | | | 95.9(3245) | | | 90.4(8368) | | | 91.6(7716) | | 94.5(5056) | | 90.2(3552) |  |  |  |
|  | Treated in 2nd. visit | 5.5(244) | | | 4.0(136) | | | 5.6(516) | | | 7.0(588) | | 5.0(266) | | 8.5(336) |  |  |  |
|  | Treated in 3rd. visit | 0.4(16) | | | 0.1(4) | | | 2.5(232) | | | 1.2(99) | | 0.5(29) | | 1.0(39) |  |  |  |
|  | Treated in 4th. visit | 0.0(2) | | |  | | | 1.2(109) | | | 0.2(17) | | 0.0(1) | | 0.2(9) |  |  |  |
|  | Treated in 5th. visit or later |  | | |  | | | 0.3(29) | | | 0.0(3) | |  | | 0.0(1) |  |  |  |
|  | Unknown | 0.0(1) | | |  | | | 0.0(3) | | |  | |  | |  |  |  |  |
|  |  |  | | |  | | |  | | |  | |  | |  |  |  |  |
| **Household Level Drug Distribution Measures** | | **N=3896** | | | | **N=3365** | **N=6471** | | | **N=5898** | | **N=453** | | **N=3680** | |  |  |  |
|  |  |  | | |  | | |  | | |  | |  | |  |  |  |  |
| *Household level Gender Community* | | | | | | | | | | | | | | | |  |  |  |
| Head of Household (HOH) Gender |  | |  | |  | | |  | | |  | |  | |  |  |  |  |
|  | Female | | 40.9(1593) | | 38.8(1304) | | | 18.0(1162) | | | 19.7(1164) | | 37.6(1707) | | 32.2(1184) |  |  |  |
|  | Male | | 56.5(2200) | | 55.5(1869) | | | 80.7(5219) | | | 78.7(4644) | | 60.0(2723) | | 55.9(2057) |  |  |  |
|  | Unknown | | 2.6(103) | | 3.9(131) | | | 1.3(85) | | | 1.5(89) | | 1.6(72) | | 11.5(424) |  |  |  |
|  | | | | | | | | | | | | | | | |  |  |  |
| Household Consent Status in Male HOH Houses |  | |  | |  | | |  | | |  | |  | |  |  |  |  |
|  | Located, household consented | | 99.2(2182) | | 87.1(1627) | | | 98.9(5163) | | | 98.3(4566) | | 94.7(2578) | | 87.8(1807) |  |  |  |
|  | Located, members not present | | 0.8(17) | | 7.2(134) | | | 0.9(47) | | | 1.5(68) | | 3.0(81) | | 5.7(118) |  |  |  |
|  | Visit attempted, not located | | 0.0(1) | | 2.8(52) | | | 0.1(3) | | | 0.1(4) | | 1.3(36) | | 3.6(74) |  |  |  |
|  | Located, refused consent | |  | | 2.9(55) | | | 0.1(5) | | | 0.1(5) | | 1.0(28) | | 2.8(58) |  |  |  |
|  | Visit was not attempted | |  | | 0.1(1) | | | 0.0(1) | | | 0.0(1) | |  | |  |  |  |  |
|  | | | | | | | | | | | | | | | |  |  |  |
| Household Consent Status in Female HOH Houses |  | |  | |  | | |  | | |  | |  | |  |  |  |  |
|  | Located, household consented | | 98.9(1576) | | 88.7(1156) | | | 97.3(1131) | | | 97.3(1132) | | 96.2(1642) | | 90.8(1075) |  |  |  |
|  | Located, members not present | | 0.9(15) | | 6.7(87) | | | 1.6(19) | | | 2.4(28) | | 2.2(38) | | 3.6(43) |  |  |  |
|  | Visit attempted, not located | |  | | 1.6(21) | | | 0.4(5) | | | 0.2(2) | | 0.9(16) | | 2.9(34) |  |  |  |
|  | Located, refused consent | | 0.1(2) | | 3.1(40) | | | 0.3(4) | | |  | | 0.6(11) | | 2.7(32) |  |  |  |
|  | Visit was not attempted | |  | |  | | | 0.3(3) | | | 0.2(2) | |  | |  |  |  |  |
|  | | | | | | | | | | | | | | | |  |  |  |
| Among Consented Male HOH Houses |  | |  | |  | | |  | | |  | |  | |  |  |  |  |
|  | Entire house treated in 1st. visit | | 81.5(1778) | | 79.3(1291) | | | 69.4(3583) | | | 73.1(3340) | | 73.9(1904) | | 58.8(1062) |  |  |  |
|  | Entire house treated in 2nd. visit | | 15.4(335) | | 16.3(266) | | | 16.4(848) | | | 20.5(935) | | 19.7(509) | | 32.3(583) |  |  |  |
|  | Entire house treated in 3rd. visit | | 2.4(52) | | 3.5(57) | | | 8.3(431) | | | 4.7(215) | | 5.3(136) | | 8.0(144) |  |  |  |
|  | Entire house treated in 4th. visit | | 0.5(10) | | 0.6(10) | | | 4.1(212) | | | 1.3(61) | | 0.8(21) | | 0.9(16) |  |  |  |
|  | Entire house treated in 5th. visit or later | | 0.3(7) | | 0.2(3) | | | 1.7(89) | | | 0.3(15) | | 0.3(8) | | 0.1(2) |  |  |  |
|  | | | | | | | | | | | | | | | |  |  |  |
| Among Consented Female HOH Houses |  | |  | |  | | |  | | |  | |  | |  |  |  |  |
|  | Unknown | |  | |  | | |  | | |  | | 0.0(0) | |  |  |  |  |
|  | Entire house treated in 1st. visit | | 86.6(1365) | | 82.6(955) | | | 80.0(905) | | | 77.7(879) | | 81.1(1332) | | 68.7(739) |  |  |  |
|  | Entire house treated in 2nd. visit | | 11.1(175) | | 13.8(160) | | | 11.1(126) | | | 16.5(187) | | 14.7(242) | | 24.4(262) |  |  |  |
|  | Entire house treated in 3rd. visit | | 1.5(24) | | 3.1(36) | | | 5.0(57) | | | 4.3(49) | | 3.5(57) | | 5.6(60) |  |  |  |
|  | Entire house treated in 4th. visit | | 0.5(8) | | 0.3(4) | | | 2.5(28) | | | 1.0(11) | | 0.3(5) | | 1.0(11) |  |  |  |
|  | Entire house treated in 5th. visit or later | | 0.3(4) | | 0.1(1) | | | 1.3(15) | | | 0.5(6) | | 0.2(4) | | 0.3(3) |  |  |  |
|  | | | | | | | | | | | | | | | |  |  |  |
| *Mark all that applies survey question. | | | |  |  |  |  |  |  |  |  |  |  |  |  |  |  |  |
| ^1^CDD (community drug distributor), HW (health worker). | | | | | | | | |  |  |  |  |  |  |  |  |  |  |
| ^2^Grahm Sabha is in India only and involves advocacy by local leaders via a localized self-governing structure at the village or town level. | | | | | | | | | | | | | | | | | | |
| ^3^Anyone who was treated at home or elsewhere in the community and not treated at school or an Anganwadi center.  ^4^Anyone who was treated at home or elsewhere in the community and was 5yo or older and was not represented by a proxy respondent was eligible for the MDA Time/preferences questions. | | | | | | | | | | | | | | | | |  |  |
| ^5^Adult includes any individual 15 years or older, all adult males and all adult females not pregnant in their first trimester are eligible for treatment with  Albendazole | | | | | | | | | | | | | | | | | |  |
| ^6^Individuals treated includes: 1-directly observed treatment, 2-tablet left with participant, 3-tablet left at house, and 4-indivudal reported recently receiving  treatment. | | | | | | | | | | | | | | | | | |  |

|  | | | | **Benin High Coverage**  **Mean %(n)** | | | **Benin Low Coverage**  **Mean %(n)** | | |  |  |
| --- | --- | --- | --- | --- | --- | --- | --- | --- | --- | --- | --- |
|  | | | | **MDA 1**  **N=5035** | **MDA 2**  **N=5127** | **MDA3**  **N=4338** | **MDA 1**  **N= 4509** | **MDA 2**  **N=4636** | **MDA3**  **N=3748** |  |  |
|  |  | **Treatment** | | | | | | | | |  |
|  | | | |  |  |  |  |  |  |  |  |
| Treatment Status | | |  |  |  |  |  |  |  |  |  |
|  | | | Directly observed therapy | 85.5 (4306) | 80.7 (4138) | 93.8 (4068) | 74.7 (3367) | 51.1 (2369) | 85.3 (3198) |  |  |
|  | | | Individual was recently treated | 0.8 (42) | 13.9 (714) | 1.5 (63) | 1.2 (54) | 15.2 (704) | 0.6 (24) |  |  |
|  | | | Not treated | 12.8 (646) | 5.3 (274) | 4.8 (207) | 21.8 (985) | 28.9 (1338) | 10.3 (387) |  |  |
|  | | | Tablet was left at house | 0.5 (25) |  |  |  |  |  |  |  |
|  | | | Tablet was left with participant | 0.3 (16) | 0.0 (1) |  | 2.3 (103) | 4.9 (225) | 3.7 (139) |  |  |
|  | |  | | | | | | | | | |
|  | | | | **India High Coverage**  **Mean %(n)** | | | **India Low Coverage**  **Mean %(n)** | | |  |  |
|  | | | | **MDA 1**  **N=7951** | **MDA 2**  **N=8402** | **MDA3**  **N=8285** | **MDA 1**  **N=7429** | **MDA 2**  **N=7669** | **MDA3**  **N=7188** |  |  |
|  |  | **Treatment** | | | | | | | | |  |
|  | | | |  |  |  |  |  |  |  |  |
| Treatment Status | | |  |  |  |  |  |  |  |  |  |
|  | | | Directly observed therapy | 74.4 (5919) | 71.0 (5962) | 72.2 (5982) | 70.8 (5257) | 70.1 (5375) | 71.9 (5170) |  |  |
|  | | | Individual was recently treated | 15.0 (1189) | 17.8 (1496) | 19.3 (1599) | 14.6 (1088) | 15.8 (1210) | 19.2 (1380) |  |  |
|  | | | Not treated | 5.2 (412) | 9.2 (771) | 4.7 (389) | 10.4 (775) | 11.7 (897) | 6.2 (444) |  |  |
|  | | | Tablet was left at house | 3.4 (273) | 0.8 (71) | 3.7 (305) | 3.2 (236) | 1.1 (85) | 1.8 (128) |  |  |
|  | | | Tablet was left with participant | 2.0 (158) | 1.2 (102) | 0.1 (10) | 1.0 (73) | 1.3 (102) | 0.9 (66) |  |  |
|  | | | | **Malawi High Coverage**  **Mean %(n)** | | | **Malawi Low Coverage**  **Mean %(n)** | | |  |  |
|  | | | | **MDA 1**  **N=6928** | **MDA 2**  **N=7100** | **MDA3**  **N=6597** | **MDA 1**  **N=5775** | **MDA 2**  **N=6003** | **MDA3**  **N=5455** |  |  |
|  |  | **Treatment** | | | | | | | | |  |
|  | | | |  |  |  |  |  |  |  |  |
| Treatment Status | | |  |  |  |  |  |  |  |  |  |
|  | | | Directly observed therapy | 66.9 (4637) | 43.0 (3050) | 83.4 (5503) | 63.8 (3683) | 43.3 (2602) | 77.1 (4206) |  |  |
|  | | | Individual was recently treated | 1.7 (116) | 21.9 (1553) | 0.3 (21) | 0.6 (36) | 16.3 (979) | 0.4 (22) |  |  |
|  | | | Not treated | 20.1 (1391) | 21.4 (1520) | 14.3 (941) | 29.8 (1719) | 37.0 (2224) | 19.7 (1073) |  |  |
|  | | | Tablet was left at house | 0.0 (1) | 0.2 (11) | 0.3 (20) | 0.7 (38) | 0.8 (46) | 1.5 (80) |  |  |
|  | | | Tablet was left with participant | 11.3 (783) | 13.6 (966) | 1.7 (112) | 5.2 (299) | 2.5 (152) | 1.4 (74) |  |  |
