# Supplementary material for: “Our desire is to make this village intestinal worm free”: Identifying determinants of high coverage of community-wide mass drug administration for soil transmitted helminths in Benin, India, and Malawi
Source: PLoS Negl Trop Dis. 2024 Feb 6;18(2):e0011819. doi: 10.1371/journal.pntd.0011819 (PMC10846705; doi:10.1371/journal.pntd.0011819)
Supplement: S6 Appendix — (DOCX) [file pntd.0011819.s006.docx]

| **S6 Summary of key themes** | | | | |
| --- | --- | --- | --- | --- |
| **Themes & sub-themes** | **Corresponding CFIR constructs** | **Exemplary quote** | **Corresponding**  **quantitative data** | **Alignment between quantitative and qualitative data** |
| **Individual participation is influenced by the perceived trustworthiness of cMDA programs** | | | | |
| **Facilitators** |  |  |  |  |
| Adults perceived themselves to be at risk for STH and felt it was necessary to receive treatment. | Design quality and packaging, Relative advantage. | “The distribution of the intestinal medicine both to school-going children and the adults is a very welcome idea. It shows that the disease is contagious and when other group of people are not receiving while others are receiving it will not be eradicated, but now we are sure that the disease will be reduced. This disease has been here and when you go the hospital, there was no its medications but now the owners are coming to distribute here it will help to eradicate the disease once for all.” Respondent 5, Local Leaders (HCC), Malawi | No corresponding quantitative data. | N/A |
| Treatment was perceived to be safer if the program was associated with a non-governmental organization rather than a government program. | Innovation source. | “Some people think negatively on these issues, mostly they say why the government is giving us this medicine. It happens that families do receive medicine but giving to children only and all parents do not drink. This shows that there are some doubts by the people from the government in the motive on distributing the drugs.” Respondent 2, Men (HCC), Malawi | No corresponding quantitative data. | N/A |
| Community members felt cMDA was more trustworthy when the program collaborated with local leaders to educate people and encourage participation in cMDA. | Engaging leaders, Engaging participants. | “The first people who will stay in front of the operations and in whom we will put our trust are the village chiefs. They are the first ones we want to find there. When we see the chiefs of village with his advisors who speak everyone will trust them.” Respondent 4, Local Leaders (HCC), Benin | Coverage surveys across all clusters indicate that among respondents who remember receiving albendazole only 5.4% (Benin), 0.2% (India), and 14.3% (Malawi) cited that they were made aware of cMDA ahead of time by community leaders. | Quantitative and qualitative data diverge indicating that local leaders provide program credibility but are not as influential in raising awareness about cMDA compared to family, friends and health workers. |
| Community members had more confidence in the program when they heard about it from health workers, specifically clinicians. | Engaging participants. | “Particularly, if the doctor comes, they come for an eye examination, people will listen. If an ordinary person says, they will not listen.” Respondent 1, Women (HCC), India | Coverage surveys indicate that on average participants heard about cMDA ahead of time from family and friends: 68.1% (Benin), 49.2% (India), and 33.1% (Malawi); CDDs: 39.1% (Benin), 10.4% (India), and 43.3% (Malawi); and health staff: 0.9% (Benin), 3.1% (India), and 17.4% (Malawi) | Quantitative and qualitative diverge in that more people heard about cMDA from CDDs and family and friends but would prefer to be reached by health center staff. |
| **Barriers** |  |  |  |  |
| Community members were not confident that implementers were aware of their deworming safety concerns in some settings. | Patient (community member) needs and resources. | “However, there are things that I hear from the public, that there are some who do not like to take the medication on empty stomach, foremost they eat to their heart’s content. Thus, many complain by wondering if it is only the medication that will be given to them to drink on an empty stomach. So, if we can find a solution that allows us to eat before drinking the medication that would be good.” Respondent 6, Local Leaders (HCC), Benin | No corresponding quantitative data. | N/A |
| There is stigma and mistrust around stool collection for STH prevalence surveys in many settings that negatively affected perceptions and trustworthiness of treatment during cMDA. | Trial conduct (non-CFIR code). | “People may call bad names to the officials who will be coming to distribute the medicine as feces officers…What should be done is to visit people and inform the importance of the program. But to the contrary people were just told to give their stools without being told the importance of it.” Respondent 1, Local Leaders (HCC), Malawi | No corresponding quantitative data. | N/A |
| **Findings that differentiate between high- and low-coverage clusters** | | | | |
| Directly observed therapy (DOT) during cMDA was perceived as more favorable in communities with high coverage as compared to low-coverage clusters in Benin. | Design quality and packaging. | “What is not good are the rules set for the distribution. The medication should not be required to be taken on the spot in front of the drug distributors. If the person asks you to leave the medication for them, we should accept.” Respondent 3, Cluster 26 Local Leaders (HCC), Benin | MDA treatment data in Benin indicate that 86.3% of participants in high-coverage clusters received DOT compared to 69.3% of respondents in low-coverage clusters. | Qualitative and quantitative data converge indicating that participants in low-coverage clusters found DOT less acceptable compared to participants in high coverage clusters. |
| Negative rumors and myths about cMDA treatment were more common in low-coverage clusters as compared to high-coverage clusters in Benin. | Knowledge and beliefs. | “But we heard some rumors. Some say they have refused treatment because they don’t know if it is to kill people.” Respondent 4, Women (LCC), Benin | MDA treatment data in Benin indicate that among adult females and males who were not treated, a larger percentage were not treated due to refusals in low vs high coverage clusters (5.5% vs 0.7% among adult males and 3.6% vs 0.9% among adult females). | Quantitative and qualitative data converge indicating that negative rumors and myths have an impact on people’s acceptance of treatment. |
| Participants in high-coverage clusters reported more positive experiences with previous community-wide health programs than participants in low-coverage clusters in Benin. | Knowledge and beliefs. | “Like measles vaccination, none of this is bad. These initiatives are aimed at saving ourselves and we thank the promoters.” Respondent 4, Men (HCC), Benin | No corresponding quantitative data. | N/A |
| **Individual willingness to participate in cMDA is influenced by a perception of convenience** | | | | |
| **Facilitators** |  |  |  |  |
| Door-to-door delivery removed previous time and cost barriers to make deworming treatment more accessible to communities. | Design quality and packaging, non-CFIR code behavioral control. | “The door-to-door initiative has helped those who can’t afford to reach to schools and hospitals or even where such drug distribution events are happening especially the elderly and children and it assures the parents that such drugs cannot be taken without eating.” Respondent 5, Men (HCC), Malawi | Coverage survey data across all clusters indicates that 97.1% (Benin), 86.2% (India), and 77.4% (Malawi) preferred door-to-door distribution over school-based or centralized distribution. | Qualitative and quantitative data converge, in that there is high demand for deworming amongst adults and door-to-door delivery methods are acceptable. |
| **Barriers** |  |  |  |  |
| Participants were dissatisfied with implementation plans that favored in-person treatment (3 attempts to treat in-person before drugs could be left for absent household members). | Design quality and packaging, intervention complexity. | “There is something that the agents do that is not right, and it is that I, the father, have taken some, my wife too, my children were not present at home; when I ask to be given their share so that I could give it to them when they return, they refused, saying that the children should be drinking it in front of them.” Respondent 2, Men (HCC), Benin | MDA treatment data indicate that the proportion of community members who were ultimately treated but could not be reached by DOT after three visits was minimal in both HCCs: 0.5% (Benin), 2.6% (India), and 0.2% (Malawi); and LCCs: 2.0% (India) and 1.0% (Malawi) (no data for Benin). | Quantitative and qualitative data diverge indicating that implementation requirements that individuals be treated via DOT and not have drugs left for them did not prohibit people from ultimately being treated. |
| **Findings that differentiate between high and low-coverage clusters** | | | | |
| Participants in low-coverage clusters expressed that cMDA was more disruptive to their day-to-day activities than in high-coverage clusters in Malawi. | Intervention complexity | “Some people may answer to say, what about our businesses? Our work? Our farms? If they don’t find us, can’t they just leave the drugs on our porches?” Respondent 1, Women (LCC), Malawi | Coverage survey data in Malawi indicate that 15.5% of respondents in high-coverage clusters believed that MDA was delivered at an inconvenient time compared to 5.5% of respondents in low-coverage clusters. | Quantitative and qualitative data diverge indicating that cMDA was disruptive only to a small percentage of people in low-coverage clusters. |
| **Individual perceptions of cMDA are shaped by overarching community acceptability over time** | | | | |
| **Findings that differentiate between high and low-coverage clusters** | | | | |
| Participants in high-coverage clusters became more receptive to the program following at least one round of cMDA compared to participants in low-coverage clusters in Malawi. | Implementation climate | “During the first MDA, a lot of people drunk the medicine because they were telling them that those who want to drink, should be drinking right here. But this second distribution, a lot of people were escaping because they knew the people who were distributing the medicine so they could run away from them.” Respondent 2, Women (LCC), Malawi | MDA coverage shows that, on average, DOT increased between MDA 1 and MDA 3 in both HCCs and LCCs in Malawi: 66.9% up to 83.4% in HCCs and 63.8% up to 77.1% in LCCs. | Quantitative and qualitative data diverge indicating that DOT treatment increased over time and the average percentage of people not treated decreased between MDA round 1 and 2 in both HCCs and LCCs. |
| **Individual participation is influenced by existing gender dynamics in communities** | | | | |
| **Facilitators** |  |  |  |  |
| cMDA engagement and delivery strategies were more successful in reaching women than men. | Gender (non-CFIR code) | “Some men may be missed out because they will not be there at that time in the house. When it is like that, they come and give the next day. If they are not available also at that time, some people may be left out. For ladies, no one is missed out” Respondent 1, Women (HCC), India | MDA treatment data showed that more adult men missed being treated (Benin: 14.9%, India: 8.1%, and Malawi: 32.2%) than women (Benin: 13.2%, India: 6.9%, and Malawi: 15.7%). | Quantitative and qualitative data converge indicating that cMDA was more successful in reaching women than men. |
| **Findings that differentiate between high and low-coverage clusters** | | | | |
| Women in high-coverage clusters were more likely to have the latitude to exert decision-making authority on behalf of their household than women in low-coverage clusters in Benin. | Gender (non-CFIR code) | “[Woman 9] said that the agents had come for the first time and her husband had refused. If it were me, when the drug distributors came and had explained to her that the drugs were intended to save our lives if he is still refusing, I would take it, because they cannot send us a deadly product to take. Even when he refused for the first time in the presence of the agents, I would challenge his attitude before them...” Respondent 3, Women (HCC), Benin | Combined census and MDA treatment data indicated that entire households were treated during the first visit more frequently in female-led households in high-coverage clusters (86.6%) compared to female-led households in low-coverage clusters (82.6%) in Benin. | Quantitative and qualitative data converge indicating that women in high-coverage clusters were able to exert more decision-making authority than women in low-coverage clusters. |
